# Assessing Contributions of Synthetic Musk Compounds from Wastewater Treatment Plants to Atmospheric and Aquatic Environments

**DOI:** 10.1021/acs.est.4c00840

**Published:** 2024-03-11

**Authors:** Wen-Long Li, Chubashini Shunthirasingham, Fiona Wong, Shirley Anne Smyth, Artur Pajda, Nick Alexandrou, Hayley Hung, Chun-Yan Huo, Tommy Bisbicos, Mehran Alaee, Grazina Pacepavicius, Chris Marvin

**Affiliations:** † Air Quality Processes Research Section, Environment and Climate Change Canada, 4905 Dufferin St, Toronto, ON M3H 5T4, Canada; ‡ Science and Risk Assessment Directorate, Environment and Climate Change Canada, Burlington, ON L7S 1A1, Canada; § Water Science and Technology Directorate, Environment and Climate Change Canada, Burlington, ON L7S 1A1, Canada; ∥ College of the Environment and Ecology, Xiamen University, Xiamen 361005, China

**Keywords:** synthetic musk compounds, wastewater, emission, chemical fate, modeling

## Abstract

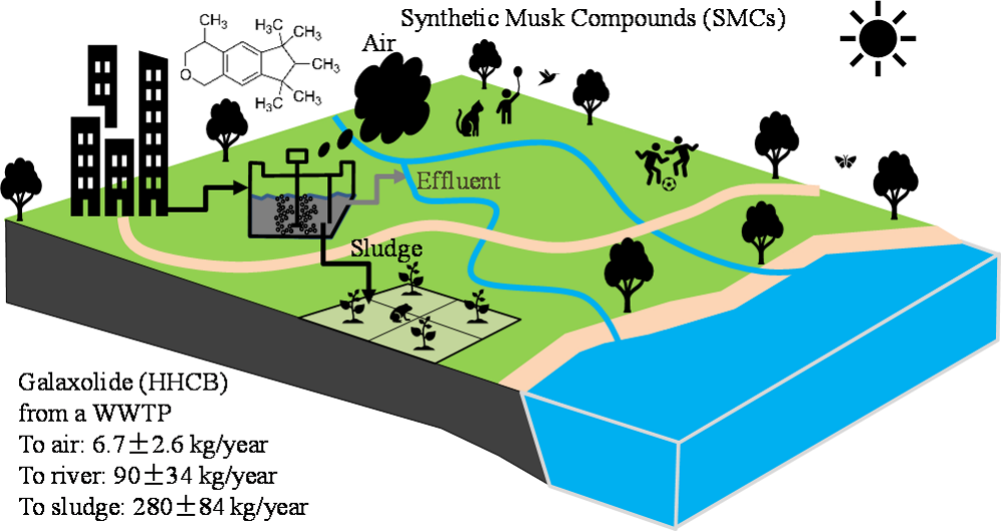

The high environmental concentrations, persistence, and toxicity of synthetic musk compounds (SMCs) necessitate a better grasp of their fate in wastewater treatment plants (WWTPs). To investigate the importance of WWTPs as pathways of SMCs to the environment, air and wastewater samples were collected at four WWTPs in Ontario, Canada. Polycyclic musks (PCMs) were present at higher concentrations than nitro musks (NMs) and macrocyclic musks (MCMs). Three PCMs [galaxolide (HHCB), tonalide (AHTN), and iso-E super (OTNE)] were the most abundant compounds (0.30–680 ng/m^3^ in air, 0.40–15 μg/L in influent, and 0.007–6.0 μg/L in effluent). Analyses of multiyear data suggest that risk management measures put in place have been effective in reducing the release of many SMCs into the environment. The highest removal efficiency, up to almost 100% of some SMCs, was observed for the plant with the longest solid retention time. A fugacity-based model was established to simulate the transport and fate of SMCs in the WWTP, and good agreement was obtained between the measured and modeled values. These findings indicate that the levels of certain SMCs discharged into the atmospheric and aquatic environments were substantial, potentially resulting in exposure to both humans and wildlife.

## Abstract 2

While effective risk management measures have reduced SMC releases, our study highlights the specific environmental discharge of certain SMCs from WWTPs, which could potentially pose exposure risks to humans and wildlife.

## Introduction

Synthetic musk compounds (SMCs) are widely used as fragrances in personal care products such as body lotions, deodorants, and hair products and in household products such as laundry detergent and fabric softener.^1−3^ As a result, SMCs are ubiquitous in the environment and have been found in various environmental media such as ambient air,^4,5^ water,^6,7^ sediments,^8^ wastewater,^9^ sludge,^10^ aquatic biota,^11^ and humans.^12,13^ While SMCs are produced to replace the expensive natural musks, SMCs are not structurally or chemically similar to the natural ones.^14^ SMCs are semivolatile chemicals with physical-chemical properties similar to some persistent organic pollutants (POPs).^14^

Three major classes of SMCs were investigated in this study: nitro musks (NMs), polycyclic musks (PCMs), and macrocyclic musks (MCMs). As the most widely used NMs,^2^ musk ketone (MK) and musk xylene (MX) were first detected in the aquatic environment and biota in the Tama River, Tokyo, Japan in 1981.^15^ NMs have shown to be weakly estrogenic and are not genotoxic by themselves, but they may increase the genotoxicity of other chemicals.^16^ The use of MX has been banned since 2011 in the European Union because of its persistent, bioaccumulation, and toxic (PBT) properties.^16^ Musk ambrette (MA) was also used for many years in consumer products, but it was discontinued due to its neurotoxic effects on organisms.^17^ PCMs were first identified in water and fish in the Ruhr River and wastewater treatment plants (WWTPs) in 1994.^18^ As a major PCM, HHCB continues to be a prevalent ingredient in numerous cosmetic products with annual production of several thousand tons.^19^ A study demonstrated that concentrations of HHCB and AHTN similar to those measured in the environment can result in oxidative and genetic damage in certain aquatic organisms.^20^ PCMs have weak estrogenic activity in humans and weak antiestrogenic activity in fish.^21^ MCMs are chemically similar to natural musks and are biodegradable;^22^ therefore, they are considered to have less potential for environmental impact than the PCMs and NMs. The MCMs are not as widely used in consumer products as PCMs because the cost of their production is very high. Therefore, the release of certain SMCs into the environment is a concern due to their extensive usage and PBT properties.

SMCs in the air represent an inhalation route for human exposure, and analyzing SMCs in the air helps to understand their distribution and the potential for deposition in areas far from the source. However, a very limited number of studies are available on the measurements of SMCs in ambient air,^23^ which may result in some regulatory gaps due to insufficient relevant studies. Kallenborn et al.^24^ reported MX, MK, HHCB, and AHTN in indoor and outdoor air samples from Norway. Xie et al.^25^ reported the occurrence of HHCB and AHTN in the Arctic atmosphere, demonstrating that HHCB and AHTN may undergo long-range transport. However, Villa et al.^26^ used a multimedia model to investigate the atmospheric transport of HHCB and AHTN, which can be classified as chemicals with high persistence and low long-range atmospheric transport. Ramirez et al.^27^ determined 5 PCMs and 3 NMs in urban and suburban air in Spain. PCMs and NMs in air in the Great Lakes region were reported previously.^28,29^ Wong et al.^4^ is the first study to report MCMs in ambient air samples collected during 2007–2014.

The major pathway for SMCs entering the environment is through their use in products and, subsequently, through wastewater. SMCs entering WWTPs are not completely removed from the effluent. Because of the lipophilic nature of SMCs, they partition onto sludge solids during the treatment steps.^30^ The sewage sludge is treated and sometimes used as an agricultural fertilizer. Therefore, the pathway of SMCs entering the atmosphere includes volatilization from WWTPs, biosolid-amended soils, landfills, and the use of products containing musk compounds. Wong et al.^4^ and Upadhyay et al.^31^ reported high levels of some SMCs in the air of WWTPs, indicating that WWTPs can be an important pathway of SMCs to the atmosphere. Our previous study investigated levels of SMCs in influent and effluent from four WWTPs in Ontario, Canada, in 2003–2004,^32^ without measuring the particle and gas phase emissions of SMCs to the atmosphere from the plants. Therefore, the major objectives of this study are (i) to assess changes in concentrations of PCMs, NMs, and MCMs in air, influent, and effluent samples from the same WWTPs at different times to determine how their usage and releases have changed over the past decade in Canada and (ii) to assess the contribution of SMCs from WWTPs to atmospheric and aquatic environments using a fugacity-based mass balance model.

## Experimental Procedure and Modeling

### WWTP Operation Notes

In this study, wastewater was collected from four different WWTPs consisting of three different types of biological treatments [an aerated lagoon, two conventional activated sludge plants (CAS 1 and CAS 2), and an oxidation ditch (OD)]. WWTPs were operating normally during all sampling periods. Plants were operated to achieve partial nitrification (CAS 2 and lagoon in the winter sampling period and CAS 1 in the summer) and full nitrification (lagoon and CAS 2 in the summer sampling period and OD during both sampling periods) using an appropriate dissolved oxygen level and solid retention time (SRT). The physical characteristics of the influent and effluent water, namely, total suspended solids (TSS), biological oxygen demand (BOD), chemical oxygen demand (COD), and total Kjeldahl nitrogen (TKN), are reported in Table S1. Typical levels of BOD and TSS were observed for all four plants (Table S1). The removal rate for TSS and BOD ranged from 79 to 98% and 87–98%, respectively.

### Sample Analyses and QA/QC

Details on the air and wastewater sample collections, analyses, and QA/QC are described in the Supporting Information (SI). In general, on-site and off-site air samples were collected at four WWTPs with different community sizes and residential inputs during the winter and summer of 2017 to study levels and seasonal variability of the SMCs in Canadian WWTPs. “On-site” samples refer to the samples collected directly within the premises of the WWTPs, allowing us to assess the concentrations of SMCs directly at the source. In contrast, “off-site” samples refer to those collected ∼100–150 m away from the WWTPs, which helps in evaluating the dispersion and distribution of SMCs in the broader environment. The municipalities served by the four WWTPs included rural and small urban areas (lagoon and oxidation ditch) and large urban areas (CAS 1 and CAS 2, see Table 1). The vapor-phase and particle-phase air samples were separately extracted by Dionex ASE350 and analyzed by an Agilent 7000C triple quadrupole mass spectrometer (MS) connected to a 7890 gas chromatograph (GC). The 21 SMCs include five NMs [musk tibetene (MT), MA, musk moskene (MM), MK, MX], and eight PCMs [1-methyl-alpha-ionone (1MAI), Cashmeran (DPMI), Iso E super (OTNE), Celestolide (ADBI), Phantolide (AHMI), Traseolide (ATII), HHCB, AHTN], and eight MCMs [exaltone (EXN), muscone (MCN), exaltolide (EXL), ambrettolide (AMB), 16-hexadecanolide (16-H), MUSK MC-4 (MC-4), cervolide (CER], ethylene brassylate (EtB)] (Table S2).

**Table 1:** Characteristics of Wastewater Treatment Plants where Air and Water Samples Were Collected^a^

WWTP type	aerated lagoon	oxidation ditch	CAS 1	CAS 2
served population^b^	1366	1988	105,000	233,000
average flow (m^3^/day) Feb/Mar 2017	510	2249	69,803	85,318
average flow (m^3^/day) August 2017	510	1037	37,889	58,705
% residential inputs	90	90	85	60
% industrial–commercial–institutional inputs	10	10	15	40
influent temperature (°C) Feb/Mar 2017	10	9–11	9–12	11–14
influent temperature (°C) August 2017	20–21	17–18	19–20	16–20
effluent temperature (°C) Feb/Mar 2017	5–6	8–9	9–12	12
effluent temperature (°C) August 2017	20–21	18	19–20	21
HRT	3–6 months	18 days	14 h	23 h
SRT	N/A	Weeks	5–7 days	2–6 days

a N/A = not available.

b Based on the 2016 census.

The 24 h composite samples of influent and treated effluent from the same WWTPs were collected concurrently as described previously.^33^ Samples were extracted by liquid–liquid extraction following the method developed by Lee et al.^34^ NMs were analyzed by GC-triple quadrupole MS in negative chemical ionization mode, and PCMs and MCMs were analyzed by GC-triple quadrupole MS in the EI mode.

### Probability Mass Balance Modeling

A fugacity-based analysis^35,36^ of the transport and fate of SMCs in WWTPs was performed. Air, influent, primary settling tank, aeration tank, secondary settling tank, and effluent were compartments included in the fugacity model (Figure S1). The environmental processes, including advection, phase partitioning, volatilization, and transformation, were considered. Fugacity (*f*, in Pa) was used as a surrogate for concentrations of *i*th SMC in the *j*th compartment (*C*_*ij*_, mol/m^3^),
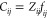
1where *Z*_*ij*_ is the fugacity capacity (mol m^3^/Pa) and is specific to temperature, the compound, and the phase in which the compound resides.

An important step of the modeling is to derive *D* values (mol/(Pa)/h), which are transport or transformation parameters. When multiplied by a fugacity, they give rates of transport or transformation. They are thus similar in principle to fugacity rate constants, with faster processes having larger *D* values.

Using the mass balance approach, the input flux (*I*) of each SMC should be equal to the output flux (*O*) in each compartment,
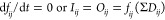
2where *f*_*ij*_ is the fugacity of a compound *i*th in a compartment *j*, and Σ*D*_*ij*_ is the sum of the *D* values for all the possible processes in the *j*th compartment.

Probability mass balance (PMB) modeling^37^ is a powerful tool to quantify and predict the fate and transport of contaminants in the WWTP. Monte Carlo simulation was utilized to simulate the uncertainty and variability of each input parameter. Monte Carlo simulation is a more flexible and powerful method than point estimation as it allows for the estimation of the entire probability distribution of the parameter, rather than just a point estimate. In this study, the model was run 10000 times to simulate the probabilistic estimate of model outputs. More details for the model (equations and parameters) can be found in the SI.

## Results and Discussion

### Atmospheric SMCs Were Related to Chemical Partitioning, Locations, WWTP Type, and Seasonality

#### Partitioning

Thirteen of the twenty one musk compounds analyzed were detected in the air samples from the four WWTPs. The concentrations of the SMCs in gas and particle phase samples collected at the WWTP on-site and the off-site locations are reported in Tables S3–S10. Significant fractions of these chemicals were found in the gas phase due to the high vapor pressure.^4^ Particle-bound fractions were generally less than 25%, indicating that SMCs were primarily found in the gas phase. ADBI, AHMI, MK, and EtB were the exception with the particle-bound fraction ranging from 17 to 67% in the cold season. In the winter, SMCs have a higher tendency to sorb to airborne particles due to a higher octanol-air partition coefficient at lower temperatures.^38^

#### On-Site vs Off-Site Locations

**Figure 1:**
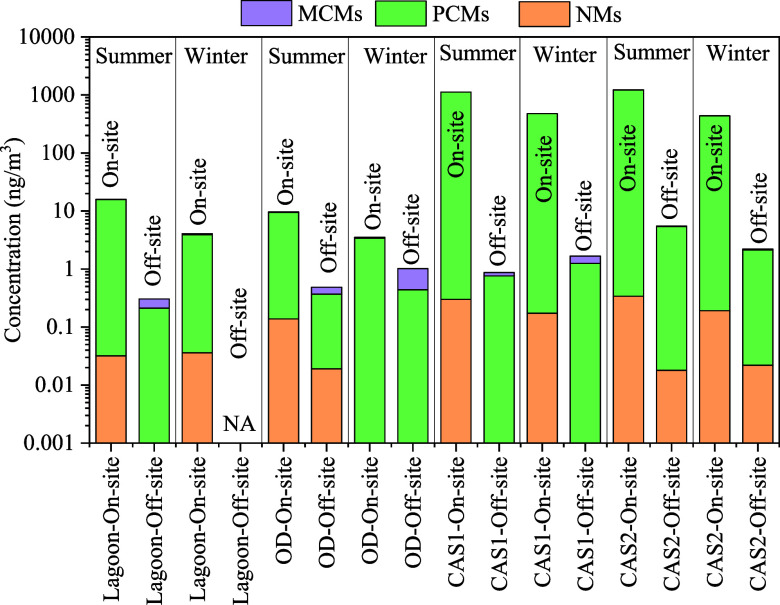
Comparisons of the concentrations (ng/m^3^) of NMs, MCMs, and PCMs in the on-site and off-site air of four WWTPs. Comparisons were performed for the (a) warm season and (b) cold season separately considering seasonal fluctuations in SMC concentrations.

The concentrations of most SMCs in the off-site air were much lower than those in on-site air (Figures 1, S2 and Tables S3–S10). The air concentrations of ΣSMCs in the off-site air ranged from 0.66 to 3.9 ng/m^3^. The air concentrations of each SMC at the off-site locations were, on average, about 8.7–630 times lower than those at the corresponding on-site locations at each WWTP, indicating emissions of SMCs from WWTPs to the atmosphere. Wong et al.^4^ also reported higher levels of some SMCs in the on-site air, compared to urban and background sites. A comparison of HHCB and AHTN concentrations measured in this study and previous studies in the USA,^14^ Canada,^28^ Norway,^24^ Spain,^27^ Arctic, and over the North Sea^25^ is displayed in Figure S3. Urban concentrations of HHCB and AHTN ranged from 1.0 to 11 ng/m^3^,^4^ and HHCB and AHTN were found in remote areas such as the Arctic at very low levels.^25^ The concentrations of HHCB plus AHTN in WWTP on-site air were on average 80 times higher than those reported in urban air, suggesting that WWTPs were important pathways for SMCs to the atmosphere.

#### WWTP Types

The concentrations of ΣSMCs in the on-site air samples collected from CAS 1 (800 ng/m^3^) and CAS 2 (830 ng/m^3^) were about 79–124 times greater than those measured at the lagoon (10 ng/m^3^) and oxidation ditch (6.6 ng/m^3^). The higher air concentrations of SMCs at CAS 2 and CAS 1 could be due to the larger served populations and greater influent loading rates by these facilities relative to the lagoon and oxidation ditch (Table 1). Therefore, the relationship between the concentrations of ΣSMCs in the on-site air and operational parameters such as served population and the influent flow rate was examined. A positive relationship was observed between the ΣSMC concentrations and the served populations or influent flow rate, suggesting that on-site air received the majority input of SMCs from emissions from the WWTP. Shoeib et al. (2016)^39^ also observed this positive relationship for poly and perfluoroalkyl substances. The concentrations of many chemicals found in WWTP influents were related to input from the served population,^40,41^ suggesting that the concentrations of SMCs in on-site air could be directly proportional to the served population.

#### Seasonality

The concentrations of SMCs in the air varied widely during the warm and cold seasons (Table S3–S10), which could be due to daily variations in the composition of wastewater and local sources. Higher concentrations of PCMs in on-site air were observed in the warm season compared to the cold season, suggesting higher emissions to the atmosphere in the warm season (Figure S4). The higher concentrations of SMCs in summer were most likely due to enhanced volatilization from WWTPs to the atmosphere as the wastewater temperature was higher in the summer.

### Loading and Removal Analyses Suggest that SMCs Were Not Completely Removed by the Studied WWTPs

Concentrations of SMCs in the influent and effluent samples (aqueous phase plus particulates) from four WWTPs are summarized in Table 2, Table S11, and Figure S5. The composition profiles of SMCs in influent samples were similar despite the different characteristics of the WWTPs. The concentrations of the ΣSMCs in influent samples ranged from 9.5 to 32 μg/L in the cold season. The highest ΣSMC concentration (32 μg/L) was measured in plant CAS 1 (in urban area), which is the second largest of the four plants, serving a population of about 105,000. The CAS 2 WWTP was the largest plant and served a population of 205000; ΣSMC concentration (27 μg/L) was lower than that in plant CAS 1 but similar to that in the plant lagoon (29 μg/L) with a smaller population of 1366.

**Table 2:** Concentrations [Mean, Standard Deviation (SD), Minimum (Min), and Maximum (Max)] of SMCs in Air (ng/m^3^) and Wastewater (ng/L) of Four WWTPs^a^

	mean	SD	min	max	mean	SD	min	max	mean	SD	min	max
	**air**	ng/m^3^			**influent**	ng/L			**effluent**	ng/L		
MA	ND	ND	ND	ND	0.38	0.75	ND	1.5	ND	ND	ND	ND
MK	0.031	0.033	ND	0.096	20	13	2.1	37	8.8	8.2	ND	19
MM	0.002	0.007	ND	0.028	0.94	0.67	ND	1.5	ND	ND	ND	ND
MT	ND	ND	ND	ND	0.64	0.79	ND	1.6	ND	ND	ND	ND
MX	0.05	0.08	ND	0.26	6.0	5.4	ND	12	4.0	3.2	ND	7.1
ΣNMs	0.079	0.11	ND	0.34	28	19	2.1	50	13	9.7	0.50	22
DPMI	0.25	0.43	ND	1.3	95	67	12	170	25	25	3.4	55
ADBI	0.12	0.18	ND	0.52	32	10	18	39	8.9	7.5	ND	16
HHCB	86	200	ND	680	12,000	63,000	3800	14,000	3200	2500	210	5800
OTNE	120	220	ND	650	11,000	5531	2700	15,000	2300	2300	26	4600
1MAI	0.09	0.25	ND	1.0	1100	690	220	1900	6.2	7.5	ND	17
AHMI	0.05	0.076	ND	0.19	ND	ND	ND	ND	ND	ND	ND	ND
AHTN	2.0	3.4	ND	11	573	120	400	680	130	96	6.9	230
ATII	ND	ND	ND	ND	31	12	13	40	6.7	6.4	1.0	14
ΣPCMs	210	410	ND	1200	24,000	10,000	9500	32,000	5600	4900	250	11,000
AMB	ND	ND	ND	ND	ND	ND	ND	ND	ND	ND	ND	ND
CER	ND	ND	ND	ND	ND	ND	ND	ND	ND	ND	ND	ND
EtB	0.09	0.102	ND	0.35	36	50	6.9	110	3.3	1.6	1.2	4.7
EXN	0.03	0.09	ND	0.27	ND	ND	ND	ND	ND	ND	ND	ND
EXL	ND	ND	ND	ND	ND	ND	ND	ND	ND	ND	ND	ND
16-H	0.051	0.036	ND	0.14	ND	ND	ND	ND	ND	ND	ND	ND
MC-4	0.031	0.027	ND	0.10	ND	ND	ND	ND	ND	ND	ND	ND
MUS	ND	ND	ND	ND	ND	ND	ND	ND	ND	ND	ND	ND
ΣMCMs	0.20	0.17	ND	0.57	36	50	6.9	110	3.3	1.6	1.2	4.7
ΣSMCs	210	410	ND	1200	24,000	10,000	9500	32,000	5600	4900	250	11,000

a ND: Not detected.

#### PCMs

PCMs were the most abundant SMCs with concentrations ranging from 9.5 to 32 μg/L in influents and 0.25–11 μg/L in effluents (Figure S5). The PCMs represent about 99% of the ΣSMCs. Same as the air samples, HHCB and OTNE were the dominant compounds in influent and effluent samples, followed by AHTN and 1MAI. Smyth et al.^32^ observed that HHCB had the highest concentration followed by AHTN for the same WWTPs, but the study did not report on OTNE. The concentrations of HHCB and AHTN were higher than those measured in a WWTP in Peterborough, Canada,^42^ and other WWTPs in Ontario, Canada,^43^ respectively. Other studies have also reported high levels of HHCB and AHTN in influent and effluent in China.^44,45^ A study showed that the PCM concentrations in WWTP vary largely among countries,^46^ which may be attributed to different consumption patterns of PCMs in personal care and household products worldwide.

#### MCMs and NMs

Most MCMs, except for EtB, were below the detection limit in the influent and effluent of all WWTPs. The concentration of EtB ranged from 6.9 to 110 ng/L in influents and from 1.2 to 4.7 ng/L in effluents. As shown in Figure S6, the ΣNMs ranged from 2.1 to 50 ng/L in influents and from 0.50 to 22 ng/L in effluents. MK and MX have the highest concentrations among the NMs and have been frequently reported in previous studies. The concentrations of NMs varied over the two sampling periods (summer and winter, Figure S6). NMs showed a trend of decreasing concentration from winter to summer, except for CAS 2. Higher concentrations of SMCs in influent collected in the winter period were likely due to the washing of heavy-duty clothing, which needed more detergent in the winter. Volatilization can be a major mechanism for the greater loss of SMCs from WWTPs due to their higher vapor pressure at warmer temperatures.

#### Loadings and Removal

The SMC concentrations in the influent were a function of the served population and the amount of SMC used. Thus, influent loadings were reflective of the populations served by each plant. The four plants varied widely in population served and flow rates (Table 1). Influent loadings per capita were calculated using influent flow rates adjusted for residential inputs, measured influent concentrations, and the populations served by each WWTP. The influent loadings for ΣSMCs were 18, 5.9, 9.7, and 9.6 mg/day/capita for plants CAS 1, CAS 2, lagoon, and OD, respectively. Plant lagoon, OD, and CAS 1 were mainly influenced by residential sewage, while CAS 2 had high industrial influences with 40% industrial, commercial, and institutional inputs. High influent loading was observed for CAS 1, which received high proportions of residential wastewater.

SMC concentrations in the effluent were lower than those in the influent, suggesting that WWTPs were effective at removing SMCs from wastewater. To examine the removal of SMCs in the four WWTPs, we calculated the removal efficiencies of eight SMCs in both the particulate and dissolved phases. As summarized in Figure S7, the removal of individual SMC ranged from 30 to 99.9% (>64% for CAS 1, >30% for CAS 2, >42% for oxidation ditch, and >96% for lagoon). The limited removal of several SMCs could potentially be attributed to the fact that WWTPs are typically not designed for the removal of SMCs. The aerated lagoon had the longest hydraulic retention time (HRT, 3–6 months) and the highest removal efficiency. This observation implies that the lagoon system provides ample opportunity for sorption, photodegradation, and volatilization processes to occur, which is particularly relevant for PCMs and NMs. These classes of SMCs are generally characterized by lower biodegradability,^23^ which means that nonbiological removal mechanisms such as sorption to sludge and volatilization into the atmosphere play a significant role in reducing their concentrations in the wastewater. Volatile and semivolatile organic pollutants can be stripped from the wastewater during the aeration process, which also promotes the formation of aerosols. Both HRT and aeration seem to play some roles in achieving better removal of SMCs in the lagoon. The removal efficiencies of SMCs in the four WWTPs were similar to those reported in 2008 for the same plants.^32^ The removal of HHCB and AHTN in this study (63–89%) was similar to those values (72–99%)^47^ for WWTPs in the USA, but higher than those (38–55%)^42^ in Peterborough, Canada. Therefore, the discharge of SMCs into the aquatic environment must be quantified.

### Multiyear Analyses of SMCs Reveal the Effectiveness of Emission Control

**Figure 2:**
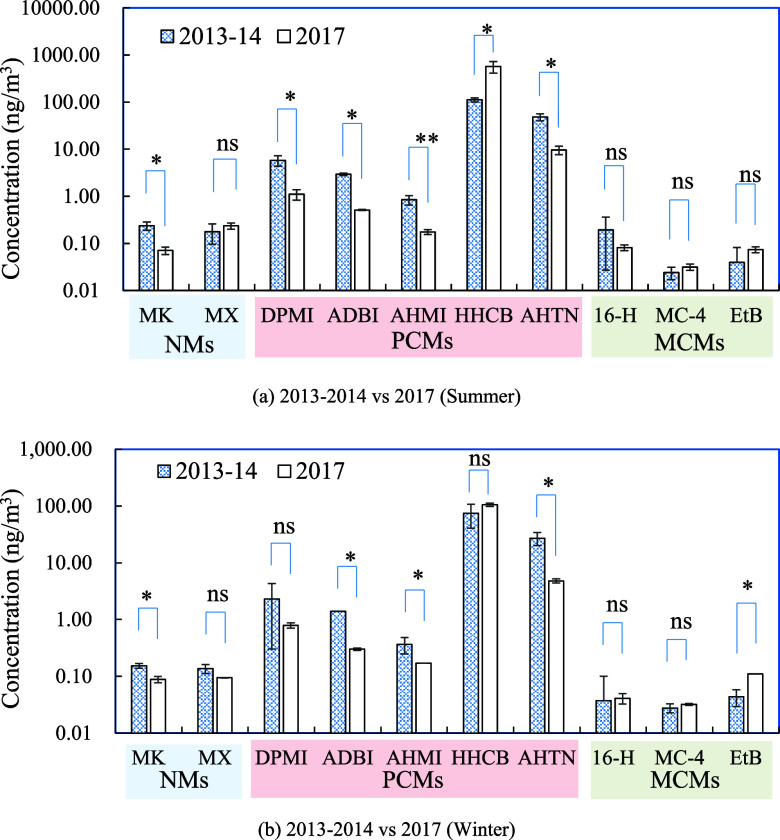
Comparisons of the SMC concentrations between two distinct sampling periods (2013–14^4^ and 2017) in on-site air. Comparisons were performed for (a) warm season and (b) cold season separately considering seasonal fluctuations in SMC concentrations. NS represents not significant; * represents difference being significant at the 0.05 level and ** represents difference being significant at the 0.01 level.

Because of the various bans and restrictions of NMs internationally due to their PBT properties,^16^ the production and use of PCMs and MCMs as substitutes could have increased. To evaluate the impact of regulations on the trends of SMCs, we first compared the SMC concentration from the on-site air collected in 2017 and those during 2013–14, which were analyzed using the same analytical methods.^4^ As shown in Figure 2, concentrations of regulated NMs, specifically MK, decreased from 2013 to 14 to 2017, indicating the effectiveness of regulatory actions on NMs. However, a major increase in HHCB concentrations was observed from 2013 to 14 to 2017 in both the summer and winter seasons. As one of the most widely used musk compounds, HHCB was listed as a high-production volume chemical by the US EPA.^48^ High concentrations of HHCB and AHTN were also reported in air of a WWTP in Arizona, USA.^31^ Concentrations of several MCMs (MC-4 and EtB) were also increasing. MCMs are chemically similar to natural musks and are biodegradable,^22^ suggesting that they may have less potential for environmental impact than the PCMs and NMs. MCMs are becoming more and more available to the market because of the advances made in synthesis methods. With a decrease in the production cost of the MCMs coupled with their environmentally friendly properties, the use of MCMs may increase in the future.

For wastewater, a similar major reduction in the levels of NMs was observed (Figure S8). Levels of MK have dropped by a factor of 8 from 2003 to 04 to 2017, while levels of MX have dropped by a factor of 26 during the cold sampling period and 130 for samples collected during the warm period. This indicates that there have been declines in the use of NMs in Canada over recent years, likely due to restrictions in other jurisdictions and a general move by the industry away from these substances, as Canada does not have risk management measures in place for SMCs. Meanwhile, implementing upgrades to WWTPs can also prove to be efficacious in mitigating the discharge of these compounds into the environment. Similar to the air samples, the concentrations of HHCB in the influent of CAS 2 (13,000 ng/L) in this study were slightly higher than those from 2003 to 04 (11,000 ng/L), while the levels of other PCMs were lower in 2017 than that of 2003–04.^32^ This could be due to a notable escalation in the production of HHCB, with figures rising from approximately 1000–4000 metric tons over the span from 2011 to 2015.^4^

### Transport and Fate Modeling Revealed High Contributions of SMCs to the Atmospheric and Aquatic Environments

#### Mass Balance

Wastewater and air samples taken from site CAS 2 in the winter season were selected to demonstrate the mass balance of the model because it represents a broad spectrum of technologies utilized in modern WWTPs and the lack of summer wastewater data for some SMCs. HHCB was selected as an example for discussion here because HHCB was the most abundant compound in the water and air samples from WWTPs. Mass balance was achieved with the total mass input and output of 0.0030 ± 0.0011 mol/h for HHCB in air, which was much lower than that in the primary settling tank (0.17 ± 0.05 mol/h), aeration tank (2.8 ± 1.0 mol/h), and secondary settling tank (2.8 ± 1.0 mol/h) (Figure S9).

#### Model Validation by Measurements

**Figure 3:**
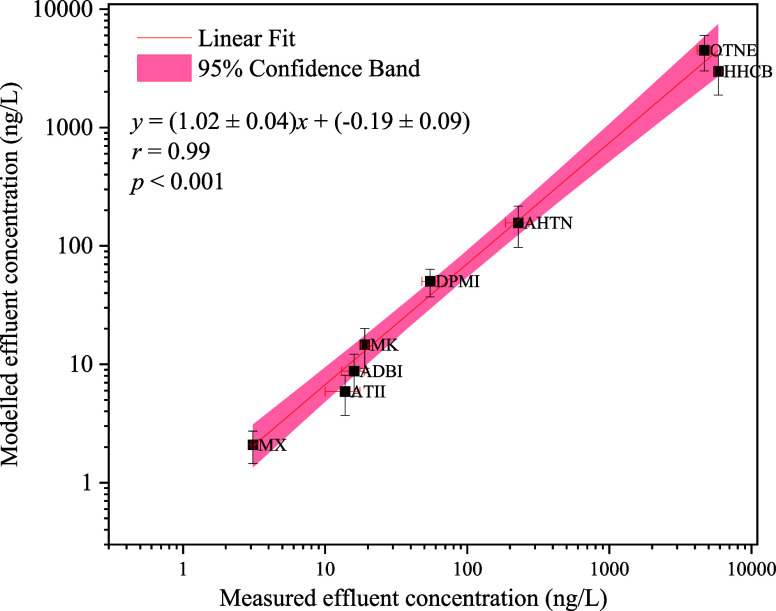
Comparison of measured and modeled effluent concentrations of SMCs.

The best way to evaluate the performance of the model is to compare the modeling results with real measurements. For wastewater effluent, the predicted concentrations of SMCs were generally comparable with that of measured concentrations within 1 order of magnitude (Figure 3), which was considered a good performance for a complex multimedia model.^49,50^ The predicted effluent concentrations and measured values were significantly correlated with each other (*r* = 0.98, *p* < 0.05) with a slope close to 1.02 and low intercept, implying a good fit between measured and estimated effluent concentrations of SMCs. For air, as shown in Figure S10, the predicted air concentrations of SMCs and the measurements were significantly correlated with each other (*r* = 0.95, *p* < 0.05) with a slope close to 1.07 and low intercept, implying a good agreement between measured and estimated values. According to the prediction, the proportion of SMCs emitted into the air ranged from 0.16 to 13.5%, which could result in considerable amounts of SMCs being re-emitted into the air because of the high inputs from wastewater influent. A cautious interpretation of these findings is still needed as the correlation does not unequivocally validate the uncertainties of the estimates. Further optimization of the parameters of some SMCs may be necessary for future studies to better predict air and effluent concentrations.

#### Transport and Fate

**Figure 4:**
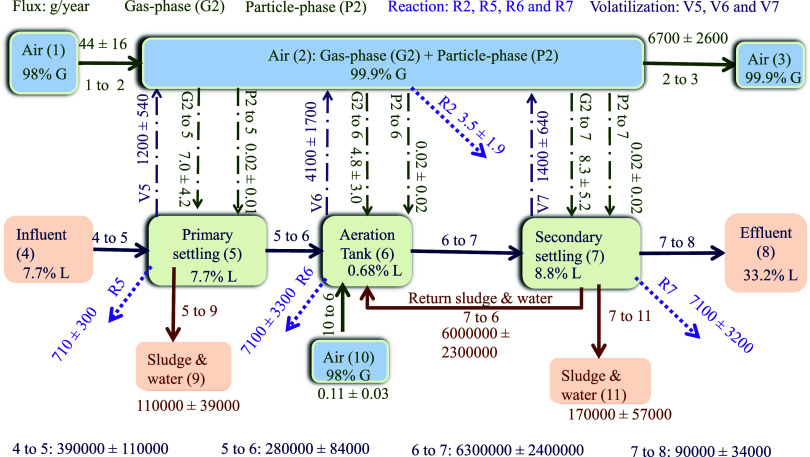
Detailed transport flux (g/year) for HHCB in CAS 2 in the winter season. The numbers in the brackets represent the compartments included in the fugacity model. Note that %G means % of HHCB present in the gas phase, and %L liquid phases represent the % of HHCB present in the liquid phase.

Detailed fluxes of advection, sorption, volatilization, and transformation of HHCB were selected as examples to demonstrate the transportation of SMCs using PMB modeling (Figure 4). Input flux from influent was calculated at 0.17 ± 0.05 mol/h or 390 ± 110 kg/year, while the output flux to effluent was 0.040 ± 0.015 mol/h or 90 ± 34 kg/year, suggesting that most of the HHCB were removed during the wastewater treatment process. The majority of HHCB was removed primarily through adsorption onto solid particles during the wastewater treatment process. Consequently, this results in an increased percentage of HHCB in the liquid phase, rising from 7.7% in the influent to 33.2% in the effluent, despite the overall removal of HHCB with the solid phase. Output fluxes to air from the primary settling tank, aeration tank, and secondary settling tank were calculated to be 1.2 ± 0.54, 4.1 ± 1.7, and 1.4 ± 0.64 kg/year, respectively, indicating that output to the air was mostly from the aeration tank. A high percentage of HHCB up to 99.9% was estimated in the gas phase, which was consistent with the measurement, indicating a predominant presence of HHCB in this phase. As air is bubbled through the wastewater in the aeration tank, semivolatile compounds are stripped from the wastewater.^51^ Output fluxes to sludge from the primary settling tank (110 ± 39 kg/year) and secondary settling tank (170 ± 57 kg/year) account for the largest proportion of input flux from influent, suggesting that most HHCB was removed via sorption to the sludge.

As shown in Figure S11, the fate of SMCs indicates that the removal of SMCs from the wastewater was governed by the combined effects of sorption, volatilization, and transformation with some proportions of SMCs remaining in the final effluent. SMCs entering WWTPs were partially partitioned to solids, with an average percentage of 63 ± 25% for all SMCs, suggesting that the removal of the majority of SMCs from wastewater occurs mainly by partitioning into sludge. More than 50% of high log *K*_OW_ SMCs, such as MM, ATII, HHCB, and AHTN, were sorbed to sludge. The MCMs had the highest proportion of removal via transformation (11–26% for different MCMs), which is consistent with the nature of MCMs as they are biologically degradable with short half-lives.^52^ Percentage contributions of SMC volatilization to the air ranged from 0.16 to 13.5%, suggesting that a significant amount of SMCs re-enter the environment through volatilization. The compounds with approximately 40% of their mass remaining in the effluent were MA, MK, MX, DPMI, and MC-4, which appeared to be due to reduced sorption and volatilization. The removal process of SMCs from WWTPs has implications for the transfer of SMCs to agricultural fields upon application of treated biosolids for fertilization. Treated biosolids are applied to agricultural land to beneficially reuse nutrients and organic carbon; therefore, the levels of SMCs in the biosolids need to be investigated as high concentrations of SMCs in biosolids could be an environmental concern.

The PMB method was used to estimate the annual contribution of SMCs to the atmospheric and aquatic environments. As shown in Figure S12, OTNE was the largest contributor to air emissions from CAS, with a contribution of 27 ± 8.1 kg/year, followed by HHCB (6.8 ± 2.6 kg/year), AHTN (3.8 ± 1.3 kg/year), and 1MAI (2.6 ± 0.71 kg/year). The contribution of these compounds to the aquatic environment was even higher, with HHCB (90 ± 34 kg/year), OTNE (130 ± 46 kg/year), 1MAI (17 ± 5.8 kg/year), and AHTN (3.3 ± 1.3 kg/year) being the dominant compounds. These amounts were significantly higher than those of parabens (0.07–7.3 kg/year) released by one WWTP effluent in China,^44^ polybrominated diphenyl ethers (1.0 kg/year) in two WWTP total discharges in Korea,^53^ and polycyclic aromatic hydrocarbon from five WWTP effluents (10 ± 4.0 kg/year) from Japan.^54^ This suggests that the release of certain SMCs, such as HHCB, from the WWTP could lead to high atmospheric concentrations, which therefore increases the risk of high exposure through inhalation.

#### Sensitive Parameters Affecting the Fate of SMCs

Sensitivity analysis is important because it helps to assess the robustness of a model’s results and predictions.^55^ The impact of changes to the values of key parameters on the overall results and predictions of SMC concentrations in the effluent and on-site air is shown in Figure S13. Taking HHCB for example, the results indicate that the HHCB concentration in the influent, *K*_OW_, TSS, particle removal, HRT, and SRT were found to have greater impacts on the effluent HHCB concentration than other parameters. This is because the HHCB concentration in the influent controls the amount of HHCB entering the WWTP, while *K*_OW_, TSS, HRT, SRT, and the particle removal control the partitioning of HHCB between the liquid and particle phases and their removal from the WWTP. The negative relationship between the concentration in the effluent and HRT/SRT indicates that longer retention times in wastewater treatment processes are generally associated with more effective removal of HHCB. This is because extended HRT and SRT provide a greater window for treatment processes to occur, which can lead to reduced concentrations of HHCB in both treated effluent and off-gassing into the air. For on-site air, there were additional important parameters that affect the modeled concentrations, such as ambient temperature, Henry’s Law constant, wind speed, and aeration rate, which control the volatilization, dispersion, and dilution of airborne HHCB. For MCMs, other parameters such as chemical half-lives could also have a strong influence on their levels because they have a higher potential for biodegradation. Off-site air concentrations of 16-H, MC-4, and EtB also showed a strong relationship with the modeled air concentrations. In conclusion, many parameters affect the transport and fate of SMCs in the WWTP and therefore impact the amount of SMCs re-entering the atmospheric and aquatic environment. Prioritizing data collection and improving the accuracy of these critical parameters is essential for achieving more accurate modeling results and therefore enhancing the reliability and usefulness of the models for assessing the environmental fate and effects of SMCs.

## Implications

This study provided the first investigations on the transport and fate of SMCs in WWTPs which were previously not well-documented in the literature. The assessment of WWTPs with varying treatment processes and operational parameters across different seasons revealed their effect on the efficacy of removing SMCs. High concentrations of SMCs were found in the influent and on-site air, suggesting that the WWTPs were significant emission pathways to the surrounding atmosphere. HHCB, AHTN, and OTNE were the most abundant SMCs found in both air and wastewater, suggesting their high usage volume, which eventually entered the WWTPs. Concentrations of SMCs in the air and wastewater were found to be significantly impacted by the ambient temperature, the treatment technology of the WWTP, and the population served, highlighting the direct influence of human activity on the release of SMCs into the environment. This reinforces the need for vigilant monitoring in areas with higher population densities. The study highlights the success of regulatory measures in reducing the concentrations of regulated NMs. However, the rise in PCMs such as HHCB suggests evolving usage patterns and production trends that warrant ongoing attention.

A fugacity-based model was established to estimate the contributions of SMCs from WWTPs to atmospheric and aquatic environments. The good agreement between measured and estimated concentrations and the sensitivity analyses on multiple parameters in our fugacity model revealed the robustness of the model’s results and predictions. Our findings indicate that WWTPs contribute to SMCs in the environment not only through the discharge of effluent and biosolids but also through direct atmospheric emissions from WWTPs. Modeled results show significant amounts of SMC sorption by sludge, which may result in exposure to terrestrial organisms when transferred to the soil from biosolids. Direct atmospheric emissions and discharge of effluents into the river system may also result in potential exposure to humans and wildlife. Concurrently, the extensive use of SMCs in personal care products results in direct human exposure to these compounds. Further studies should be carried out to gain a better understanding of the potential ecological risks and the fate of these compounds in the environment.

## Supporting Information Available

Methods and materials on chemicals, air sampling and analysis, wastewater sampling and analysis, WWTP information, QA/QC, and calculations of *Z* value, *D* value, and *f* value. Results on the comparison of the concentrations of different types of SMCs. Supporting tables on the performance of WWTPs, characteristics of WWTPs, quantifying and qualifying ions of SMCs, MRM transitions, concentrations of SMCs in air and WWTP, chemical properties of SMCs, calculated half-lives, and environmental properties. Supporting figures on the diagram of the fugacity model, SMC concentrations in the on-site and off-site air, comparisons of concentrations of HHCB and AHTN with other studies, concentrations of AHTN, HHCB, and OTNE in the air during warm and cold seasons, concentrations of PCMs, NMs and MCMs in WWTP, removal efficiencies of SMCs, comparisons of NM concentrations between 2003/2004 and 2017, mass balance of HHCB, correlations between measured and modeled air concentration, estimated proportions of different processes by the model, contributions of HHCB from WWTP and sensitivity analysis, and additional 16 references (PDF)
