# Supplementary material for: Assessing Contributions of Synthetic Musk Compounds from Wastewater Treatment Plants to Atmospheric and Aquatic Environments
Source: Environ Sci Technol. 2024 Mar 11;58(12):5524–33. doi: 10.1021/acs.est.4c00840 (PMC10976898; doi:10.1021/acs.est.4c00840)
Supplement: Supplementary file 1 — es4c00840_si_001.pdf [file es4c00840_si_001.pdf]

Supporting Information for

Assessing Contribution of Synthetic Musk Compounds from Wastewater Treatment Plants to Atmospheric and Aquatic Environments

Wen-Long Li[a, d,$], Chubashini Shunthirasingham [a,$], Fiona Wong [a], Shirley Anne Smyth[b], Artur Pajda[a], Nick Alexandrou[a], Hayley Hung[a*], Chun-Yan Huo[a, d], Tommy Bisbicos[c], Mehran Alaee[c,#], Grazina Pacepavicius[c], Chris Marvin[c]

[a] Air Quality Processes Research Section, Environment and Climate Change Canada, 4905 Dufferin St, Toronto, ON, M3H 5T4 Canada

[b] Science and Risk Assessment Directorate, Environment and Climate Change Canada, Burlington, ON, L7S 1A1 Canada

[c] Water Science and Technology Directorate, Environment and Climate Change Canada, Burlington, ON, L7S 1A1 Canada

[d] College of the Environment and Ecology, Xiamen University, Xiamen, China

55 pages, 14 tables, 14 figures and 16 references.

Corresponding author contact: +1-416-739-5944, Hayley.Hung@ec.gc.ca

[$] Equal contribution to the work.

[#] Author Information note: Date of death April 28, 2021.

---

*Corresponding author contact: +1-416-739-5944, Hayley.Hung@ec.gc.ca

Table of Contents

S1. Supplementary Methods and Results 4

S1.1. Chemicals 4

S1.2. Air Sampling and Analysis 4

S1.3. Wastewater Sampling and Analysis 6

S1.4. WWTP Upgrades 8

S1.5. Quality Control and Quality Assurance (QA/QC) 8

S1.6. Calculations of *Z* Value, *D* Value, and *f* Value 9

S1.7. Comparison of the Concentrations of Different *Types of SMCs.* 11

S2. Supplementary Tables 13

Table S1(a). Performance of the four wastewater treatment plants 13

Table S1(b). Characteristics of wastewater treatment plants where air and water samples were collected. 14

Table S2(a). Quantifying and qualifying ions for the 21 SMCs analyzed by GC-MSMS in electron ionization mode. 15

Table S2(b). Multiple Reaction Monitoring (MRM) transitions in electron capture negative ion (ECNI) and instrument detection limits (IDLs) for NMs. 15

Table S3. Concentrations [Mean (SD; Min-Max), in pg/m^3] of synthetic musk compounds in on-site and off-site air of **Lagoon** during the **summer period**. 16

Table S4. Concentrations [Mean (SD; Min-Max), in pg/m^3] of synthetic musk compounds in on-site and off-site air of **Oxidation Ditch** during the **summer period**. 17

Table S5. Concentrations [Mean (SD; Min-Max), in pg/m^3] of synthetic musk compounds in on-site and off-site air of **CAS 1** during the **summer period**. 18

Table S6. Concentrations [Mean (SD; Min-Max), in pg/m^3] of synthetic musk compounds in on-site and off-site air of **CAS 2** during the **summer period**. 19

Table S7. Concentrations [Mean (SD; Min-Max), in pg/m^3] of synthetic musk compounds in on-site and off-site air of **Lagoon** during the **winter** period. 20

Table S8. Concentrations [Mean (SD; Min-Max), in pg/m^3] of synthetic musk compounds in on-site and off-site air of **Oxidation Ditch** during the **winter** period. 21

Table S9. Concentrations [Mean (SD; Min-Max), in pg/m^3] of synthetic musk compounds in on-site and off-site air of **CAS 1** during the **winter** period. 22

Table S10. Concentrations [Mean (SD; Min-Max), in pg/m^3] of synthetic musk compounds in on-site and off-site air of **CAS2** during the **winter** period. 23

Table S11. Concentrations (Mean ± SD, ng/L) of SMCs in influent and effluent samples from four wastewater treatment plants during the winter season. 24

Table S12. Chemical properties [Log K_{OW}, vapour pressure (VP), water solubility (WS), Henry's low constant (HLC), Log K_{AW}, and Log K_{OA}] for SMCs. 25

Table S13. The values to calculate the half-lives (HL) in air, water, and biota. 26
Table S14. The environmental properties used for the model..27
S3. Supplementary Figures 28
Figure S1. Diagram of fugacity transport and process in the studied WWTP. 28
Figure S2. Average SMC concentrations (gas + particle) in the on-site (solid bars) and off-site (open bars) air samples collected at four WWTPs in Ontario, Canada in the winter and summer. 29
Figure S3. Comparisons of concentrations of HHCB and AHTN in air measured in this study with those reported in previous studies...30
Figure S4. Comparisons of the concentrations of AHTN, HHCB, and OTNE in the air of WWTPs during warm and cold seasons. 31
Figure S5. The concentrations of PCMs and MCMs in influent and effluent of four WWTPs (CAS: conventional activated sludge; OD: oxidation ditch) during the cold season. 32
Figure S6. Concentrations of NMs in influent and effluent of four WWTPs (OD: oxidation ditch, CAS: conventional activated sludge) during warm and cold seasons...33
Figure S7. Removal efficiency for SMCs in four WWTPs during the cold season. 34
Figure S8. Comparisons between levels of NMs in influent and effluent of WWTP between 2003/2004 and 2017. Comparisons were performed for warm season and cold separately considering seasonal fluctuations in SMC concentrations...35
Figure S9. Mass Balance of HHCB in the CAS 2 WWTP in the winter season. 36
Figure S10. Correlations between measured air concentration and modeled air concentration. 37
Figure S11. Estimated proportions (%) of advection, sorption, volatilization, and biotransformation of the studied SMCs in the WWTP...38
Figure S12. Contributions of HHCB from WWTPs to atmospheric and aquatic environments. 40
Figure S13. Sensitivity analysis of parameters using the correlation coefficient of each parameter and the output effluent and air concentrations for each SMC. 43
S4. References:...54

S1. Supplementary Methods and Results

S1.1. Chemicals

This study examined 21 targeted chemicals, including 5 nitro musks (NMs) which are [musk ketone (MK), musk xylene (MX), musk ambrette (MA), musk moskene (MM), musk tibetene (MT)], 8 polycyclic musks (PCMs) which are [1-methyl-alpha-ionone (1MAI), cashmeran (DPMI), Iso E super (OTNE), celestolide (ADBI), phantolide (AHMI), traseolide (ATII), galaxolide (HHCB), tonalide (AHTN)], and 8 macrocyclic musks (MCMs) which are [exaltone (EXN), muskone (MUS), exaltolide (EXL), ambrettolide (AMB), 16-hexadecanolide (16-H), musk MC-4 (MC-4), cervolide (CER), ethylene brassylate(EtB)]. These 21 standards and spiking internal standards (d_{15}-MX, d_9-MK, d_3-AHTN) were obtained from the Promochem GmbH (Wesel, Germany).

S1.2. Air Sampling and Analysis

Air samples were collected at four WWTPs in Ontario, Canada, with different community sizes and residential inputs during winter (February 28 to March 9) and summer (August 15 to 31) of 2017 to study levels and seasonal variability of the SMCs in Canadian WWTPs. At each WWTP, three on-site and one off-site air samples were obtained. The on-site samples were collected above the aeration tank or adjacent to the lagoon, while the off-site samples were collected about 100 to 150 m away from the on-site sampling location in the upwind direction of the WWTPs. The WWTPs participated in this study on the condition of anonymity, thus names and locations of each WWTP are not disclosed. The four WWTPs included one aerated lagoon, one secondary oxidation ditch, and two secondary conventional activated sludge plants. The municipalities served by the four WWTPs included rural and small urban areas (lagoon and oxidation ditch) and large urban

areas (conventional activated sludge: CAS 1 and CAS 2). Characteristics of the four WWTPs are summarized in **Table 1** and their performance is given in **Table S1** (Supporting Information).

Air sampling was achieved by a high-volume (HiVol) sampler equipped with one glass fiber filter (GFF) (10.2 ID, type A/E, Pall Laboratory) to collect particles, and PUF (Pacwill) /XAD-2 (Supelco, 15 g)/PUF sandwich (hereafter referred as PUF sandwich) to sample vapour phase. The sampling media were pre-cleaned prior to use, in which the GFF was baked at 400 °C for 24 hours, and the PUFs were extracted by an accelerated solvent extraction system, Dionex ASE350, first using acetone/hexane (1:4) twice, followed by hexane. The PUFs were dried under a gentle stream of nitrogen for 70 minutes at ~60 °C. The GFFs were wrapped in aluminum foil and stored in Ziploc bags. The PUF sandwiches were sealed in glass jars until use. Each sample represented ~540 m^3 of air, taken over 24 hours with a flow rate of ~25 m^3/hour.

All samples were stored at 4 °C until analysis. Prior to extraction, 100 ng each of d_{15}-musk xylene, d_9-musk ketone, and d_3-AHTN were spiked to each sample to monitor their recoveries. The GFFs and PUF sandwiches were separately extracted by Dionex ASE350. The extraction was carried out using hexane (100%, 3 cycles) at 75 °C, 240 s purge, static time of 5 minutes, and a rinse volume of 100%. All samples were concentrated on a Turbovap (Biotage) at 35 °C with a gentle stream of nitrogen with isooctane as keeper to a final volume of 1 mL. The extracts were passed through sodium sulfate (baked at 450 °C overnight) columns to remove water residues. Extracts were again concentrated to 1 mL using a stream of nitrogen and then solvent-exchanged into iso-octane. The final volume of the extracts was 1 mL, and 200 ng of d_{10}-fluoranthene was added to the samples as an injection standard prior to instrumental analysis.

The air sample extracts were analyzed for SMCs using an Agilent 7000C triple quadrupole mass spectrometer (MS) connected to a 7890 gas chromatograph (GC). The GC-triple quadrupole

MS was operated in multiple reaction mode under electronic ionization (EI) conditions. Chromatographic separation was carried out using a 30 m DB-5 column with a helium flow rate of 1.0 mL/min. The temperature of the transfer line, injector interface, and ion source were set at 280 °C, 270 °C, and 230 °C, respectively. The GC oven temperature was programmed with an initial temperature of 80 °C, then ramped 5 °C/min to 160 °C for 8 min, 4 °C/min to 230 °C, and 20 °C/min to 300 °C for 10 min. The monitoring ions for each SMC are reported in **Table S2**. Musk compounds were quantified against the internal standard.

S1.3. Wastewater Sampling and Analysis

Wastewater samples were collected from the same four WWTPs where air samples were collected. All samples of influent and treated effluent from the WWTPs were collected using Hach refrigerated auto-samplers (Loveland CO, USA) with Teflon-lined tubing and stainless-steel containers at a sampling frequency of 200 mL every 15 minutes to create 24-hour composite samples. Influent and effluent samples were collected concurrently, i.e., not adjusted for hydraulic retention time. This sampling method has been previously described.[1] Subsample was transferred into pre-cleaned 1L amber glass bottles with Teflon-lined lids (Systems Plus, Baden ON). All samples were stored in coolers with ice packs during transport. Upon arrival, they were stored at 4°C until analysis.

For the liquid-liquid extraction, the method developed by Lee et al. (2003)[2] was used. In this procedure, 500 mL of water or wastewater sample was spiked with internal standards (200 ng of d_3-AHTN and d_{15}-musk xylene). Then 50 mL of petroleum ether and 10 g of sodium chloride were added to each sample and stirred for 30 minutes. Each sample was then extracted a second time using 50 mL of petroleum ether. Combined organic extracts were then passed through a layer of anhydrous sodium sulfate, and evaporated with a rotary evaporator to a volume of 5 mL. Samples

were then evaporated under nitrogen in a disposable centrifuge tube to 1 mL and exchanged into iso-octane prior to clean-up using micro silica gel columns. Clean-up was accomplished using Pasteur pipettes filled with 2 g of 5% deactivated silica gel prewashed with 3 mL of hexane. The sample extract was then applied to the column. The column was first eluted with 5 mL of 1% acetone in hexane which was discarded. The musk compounds were then eluted with 10 mL of 5% acetone in hexane, evaporated, and exchanged into iso-octane. Then 50 ng of $^{13}C_6$ pentachloronitrobenzene was added to the final extract as a performance standard and the volume was adjusted to 1 mL.

All samples were analyzed using an Agilent 7000C triple quadrupole mass spectrometer connected to a 7890B GC, operated in ECNI and MRM modes. Methane was used as reagent gas at 2 mL/min, nitrogen as collision gas at 0.8 mL/min, and helium as quench gas at 2 mL/min. Two microliters of each sample were introduced into the gas chromatograph via a 7693 autosampler. The gas chromatograph was equipped with a split/splitless injector operated at 250°C. Gas chromatographic separation was accomplished using a 60 m DB5-ms (Agilent Technologies, Mississauga, Ontario) with helium at 1 mL/min constant flow as carrier gas. The oven temperature program was as follows: initial oven temperature was 70°C held for 5 min, raised to 160°C at 5°C/min, raised to 230 °C at 2.5 °C/min, and raised to 300 °C at 20 °C/min and held for 10 min.

Accurate mass determinations were accomplished using an Agilent 7200 QToF connected to a 7890B GC operated in negative ion mode. Methane and nitrogen were used as reagents and collision gases. One microliter of each sample was introduced into the gas chromatograph via a 7693 autosampler. The gas chromatograph was equipped with a split/splitless injector operated at 250°C. Gas chromatographic separation was accomplished using a 30 m HP5-MS (Agilent Technologies, Mississauga, Ontario) with helium at 1.2mL/min constant flow as carrier gas. The

oven temperature was programmed as follows: initial temperature 80°C held for 1 min, increased at 30 °C/min to 150°C, increased at 10 °C/min to 190°C, increased at 1 °C/min to 195 °C, increased at 30 °C/min to 280 °C, and held for 3 min. MS and MSMS scan ranges and acquisition rates were set to 50-500 Da and 50-300 Da, 3 Hz and 2 Hz respectively. **Table S2**(b) shows the MRM transitions for the NMs.

S1.4. WWTP Upgrades

Between the original study in 2004 and this study in 2017, infrastructure upgrades were implemented at the lagoon, CAS1, and CAS2 to improve treatment efficiency and effluent quality. Thus, nitrifying conditions of plants CAS1, CAS2, and lagoon may have improved the removal of SMCs from wastewater. The upgrades included more extensive and efficient aeration and longer solid retention time. Plants lagoon, CAS1, and CAS2 were effectively aerated using fine bubble diffusers instead of mechanical aeration. The lagoon treatment system consists of two cells instead of one cell and the plant was operated to nitrify to achieve removal of ammonia. Sand filters were also used to have better removal of solids. CAS2 was operated to achieve partial nitrification. CAS 1 utilized longer solids retention time to remove biodegradable organics, which also helps to achieve partial nitrification. A previous study has shown that the removal of trace contaminants is correlated with degree of nitrification.[3]

S1.5. Quality Control and Quality Assurance (QA/QC)

Laboratory and field blanks were processed in the same way as the samples to determine the contamination introduced during extraction and by handling, shipping, and storage, respectively. For air sampling, 2-4 field blanks were collected for each sampling season at every site by placing a sampler head with a PUF sandwich and a GFF in the HiVol sampler and immediately removing without drawing air through the sampler. Solvent blanks were included with every batch of

samples. Trace levels of OTNE, ADBI, HHCB, and EtB were found in the field (n = 15) and solvent blanks (n = 8) which were much lower than those in the air samples. The mean concentration (± standard deviations) of these chemicals in the field and solvent blanks were: OTNE 0.066 ± 0.020 ng/m^3; ADBI 0.034 ± 0.035 ng m^{-3}; HHCB 0.090 ± 0.033 ng/m^3; EtB 0.014 ± 0.023 ng/m^3. Data presented here are blank corrected. Recoveries of the deuterated musk compounds were in the range of 69-136%. For wastewater samples, the average spike recoveries for nitro musk compounds ranged between 86 ± 8% and 109 ± 5%. The sensitive method was able to detect trace amounts of nitro musk compounds in blanks. Nevertheless, the levels of nitro musk compounds in blank water samples were 0.09 ± 0.03 and 0.15 ± 0.06 ng/L, which were much lower than those in the wastewater samples.

S1.6. Calculations of *Z* Value, *D* Value, and *f* Value

Values of *Z* for each SMC in each phase can be calculated with high or low *Z* values reflecting high or low chemical concentrations expected in that phase at equilibrium. The *Z* value of a compound in the air (Z_A) is calculated as,

$$Z_A = 1/RT \quad \text{(S1)}$$

where R is the gas constant (8.314 Pa m^3/mol/K) and T is the absolute temperature in K. Z_A equal to about 4.1×10^{-4} mol/Pa/m^3 depending on the temperature. Parameters used to calculate the *Z* value and *D* value are shown in **Table S12-14**.

For compounds in water, the *Z* value (Z_W) is calculated as,

$$Z_W = 1/H \quad \text{(S2)}$$

where H is Henry's law constant (Pa m^3/mol). The temperature dependence of H for most compounds was derived from a previous study[4], and the water temperatures are shown in **Table 1**.

For compounds in solids, the Z value (Z_S) is calculated as,

$$Z_S = C_S \times Z_W / C_W \quad \text{(S3)}$$

where C_S and C_W are concentrations measured in biomass and aqueous phase that are assumed to represent equilibrium.

For advective processes such as the inflow and outflow of air, water, particles present in the air, and particles and biota present in water, the fugacity rate constant (D_{AP}) is calculated as,

$$D_{\mathrm{AP}} = G \times Z \quad \text{(S4)}$$

where G is the flow rate of the phase with the unit of m^3/h.

For degradation processes, the fugacity rate constant (D_{R}) is expressed as,

$$D_{\mathrm{R}} = k \times V \times Z \quad \text{(S5)}$$

where V is the volume of the phase (m^3), and k is the first-order rate constant (1/h) which were estimated from the EPI Suite BIOWIN model[5].

For volatilization, the D value (D_{V}) is calculated as,

$$D_{\mathrm{V}} = K_V \times A \times Z \quad \text{(S6)}$$

where K_{V} is the overall mass transfer coefficient (m/h), *and A* is the interfacial area of air and water (m^2). K_{V} is related to the waterside (K_W), airside mass transfer coefficients (K_A), and the dimensionless Henry's law constant (H'),

$$1/K_V = 1/K_W + 1/K_A H' \quad \text{(S7)}$$

Using the mass balance approach, input flux (I) should be equal to output flux (O) in each compartment,

$$I = O = f(\Sigma D) \quad \text{(S8)}$$

where f is the fugacity of a compound in a compartment, and ΣD is the sum of the D values for all the possible processes in that compartment. The chemical transport and transformation processes for each compartment of the studied WWTP are shown in **Figure S1**.

A diagram of fugacity transport and process in the studied WWTP is shown in **Figure S1**. The differential equations for the compartments (air, primary settling tank, aeration tank, secondary settling tank) in the model are derived from the steady-state mass balance equations:

$$\text{Air (2): } f_1 \times D_{12} + f_5 \times D_{V5} + f_6 \times D_{V6} + f_7 \times D_{V7} = f_2 \times D_{T2} \quad \text{(S9)}$$

$$\text{Primary settling tank (5): } E_4 + f_2 \times D_{25} = f_5 \times D_{T5} \quad \text{(S10)}$$

$$\text{Aeration tank (6): } f_5 \times D_{56} + f_2 \times D_{26} + f_7 \times D_{76} + f_1 \times D_{106} = f_6 \times D_{T6} \quad \text{(S11)}$$

$$\text{Secondary settling tank (7): } f_6 \times D_{67} + f_2 \times D_{27} = f_7 \times D_{T7} \quad \text{(S12)}$$

The numbers in the brackets or the subscript represent the compartments included in the fugacity model. The subscript "V" and "R" represent the volatilization and reactions in the compartment. The subscript "T" represents the total D value in the compartment. The f values can be calculated by solving these equations.

All the calculations were performed by using the probability approach, i.e., the Monte Carlo simulation. The basic idea behind Monte Carlo simulation is to simulate the behavior of a system or process, using random input values that reflect the uncertainty and variability of the system. The simulation generates a large number of possible outcomes, which are used to estimate the probability distribution of the system's output. This distribution can be used to estimate the likelihood of certain outcomes. For sensitivity analysis, a 20% uncertainty was assumed for the input parameters.[6, 7]

S1.7. Comparison of the Concentrations of Different Types of SMCs

PCMs. PCMs were the most abundant SMCs in air for both on-site and off-site locations at the two CAS WWTPs (**Figure 1**), accounting for 97-99% and 50-97% of the Σ_{13}SMCs in on-site and off-site air, respectively. At the lagoon and oxidation ditch, concentrations of OTNE (3.9 and 2.8 ng/m^3) and HHCB (5.1 and 3.0 ng/m^3) were similar, which were about nine times higher than AHTN (0.56 and 0.37 ng/m^3). At CAS 1 and CAS 2, the highest on-site concentrations of PCMs were observed for OTNE (430 and 510 ng/m^3) > HHCB (290 and 390 ng/m^3) >AHTN (6.3 and 8.1 ng/m^3) (**Figure S2**).

NMs and MCMs. NMs contributed very little to the SMCs (**Figure 1**). Lagoon and oxidation ditch WWTPs showed similar levels of MCMs and PCMs in off-site air (**Figure 1**), perhaps off-site air samples were not far enough away from the wastewater tanks to obtain true background levels. Only two NMs (MX and MK) were mainly detected in on-site air at very low concentrations (**Figures 1** and **S2**) and the levels ranged from ND to 0.18 ng/m^3. Four MCMs such as EtB, EXLT, 16-H, and MC-4 were detected in air and the levels were similar in on-site and off-site air. The total levels of the four MCMs ranged from 0.13 to 0.3 ng/m^3 in on-site air and 0.080 to 0.35 ng/m^3 in off-site air. Air concentrations of Σ_4MCMs were similar between the on-site and off-site air for CAS 1. Furthermore, ΣMCMs were significantly higher ($p < 0.05$) in the off-site air than in the on-site air at the lagoon and oxidation ditch. This may indicate an additional source of MCMs (other than the WWTP) at this off-site sampling location.

S2. Supplementary Tables

Table S1(a). Performance of the four wastewater treatment plants

Parameter	Type	Season	Lagoon	CAS2	Oxidation Ditch	CAS 1
Biochemical Oxygen Demand (BOD), mg/L	Influent	Winter	110 – 164	152 – 200	86 – 246	117 – 420<br>194 – 324
		Summer	210 – 228	222 – 294	177 – 219	138 – 202
	Effluent	Winter	7.8 – 9.3	18 – 20	4.2 – 5.4	7.2 – 8.1<br>12 - 14
		Summer	5.1 – 15	5.1 – 6.9	4.5 – 4.9	5.9 – 10
	% Removal	Winter	93 – 95	87 – 91	95 – 98	93 – 98<br>93 – 96
		Summer	93 – 98	97 – 98	97 – 98	93 – 97
Chemical Oxygen Demand (COD), mg/L	Influent	Winter	256 – 454	430 – 492	167 – 896	217 – 732<br>437 – 873
		Summer	554 – 715	462 – 786	419 – 672	224 – 628
	Effluent	Winter	82 – 107	66 – 80	26 – 31	55 – 70<br>61 – 65
		Summer	99 – 124	41 – 54	34 – 43	48 – 72
	% Removal	Winter	68 – 81	84 – 86	84 – 96	75 – 90<br>86 – 93
		Summer	81 – 83	89 – 93	90 – 95	68 – 92
Total Suspended Solids (TSS), mg/L	Influent	Winter	60 – 164	225 – 453	79 – 807	143 – 500<br>504 – 867
		Summer	277 – 430	215 – 336	258 – 1220	250 – 500
	Effluent	Winter	6.7 – 33	9.7 – 18	< 5.0	< 5.0 – 5.9<br>13 – 17
		Summer	< 5.0 – 5.7	< 5.0	< 5.0	< 5.0 – 6.8
	% Removal	Winter	79 – 96	94 – 98	> 94	> 96<br>97 – 98
		Summer	> 98	> 98	> 98	> 97
pH	Influent	Winter	7.70 – 7.78	7.49 – 7.61	7.54 – 7.64	7.60 – 7.69<br>7.66 – 7.75
		Summer	8.25 – 8.37	7.59 – 7.67	7.51 – 7.70	7.59 – 7.63
	Effluent	Winter	7.78 – 7.88	7.50 – 7.55	7.97 – 8.10	7.85 – 8.00<br>7.45 – 7.54
		Summer	7.76 – 7.92	7.66 – 7.81	8.04 – 8.07	7.45 – 7.81
Total Kjeldahl (organic) Nitrogen (TKN), mg/L N	Influent	Winter	37 – 38	34 – 42	10 - 17	27 – 40<br>55 – 79
		Summer	82 – 91	50 – 51	32 – 45	37 – 53
	Effluent	Winter	7.8 – 8.6	8.7 – 13	0.74 – 0.88	21 – 32<br>11 – 13
		Summer	2.6 – 2.9	2.3 – 4.9	0.99 – 1.3	1.9 – 9.7
	% Removal	Winter	77 – 80	62 – 79	91 – 96	21 – 23<br>78 – 85
		Summer	96 – 97	90 – 96	97 – 98	74 – 96
Overall water treatment performance			• Summer better<br>• Nitrification in both seasons	• Summer better<br>• Nitrification in both seasons	Very wide range of influent COD and TSS<br>Very low influent TKN in winter<br>Performance is not different between seasons<br>Nitrification in both seasons	No nitrification in winter 1<br>Nitrification in summer and winter 2<br>BOD, COD and TSS removal are not different among all

Table S1(b). Characteristics of wastewater treatment plants where air and water samples were collected.

WWTP Type	Aerated Lagoon	Oxidation Ditch	CAS 1	CAS 2
Significant industries	Sandpaper Company	Restaurants, Tourism	Universities, Landfill Leachate	N/A
Operated to nitrify?	Yes	Yes	No	Yes
Phosphorus removal	Alum	Alum	Ferric	Ferric
Tertiary filtration	Sand Filters	Sand Filters	No	No
Disinfection	No	Ultraviolet	Ultraviolet	Ultraviolet
Sludge treatment type	N/A	Aerobic Digestion	Anaerobic Digestion	Anaerobic Digestion

N/A = not available.

[a] Based on the 2016 census.

Table S2(a). Quantifying and qualifying ions for the 21 SMCs analyzed by GC-MSMS in electron ionization mode.

Type	CAS no.	Chemical	Abbreviation	Quantifier precursor ion	Quantifier product ion	Qualifier precursor ion	Qualifier product ion
NMs	145-39-1	Musk Tibetene	MT	266.1	251	251.1	91
NMs	83-66-9	Musk Ambrette	MA	268.1	253	253.1	91.1
NMs	116-66-5	Musk Moskene	MM	263.1	128	278.1	263
NMs	81-14-1	Musk Ketone	MK	279.1	91	294.1	279.1
NMs	81-15-2	Musk Xylene	MX	282.1	91	297.1	282.1
PCMs	7779-30-8	1-Methyl-Alpha-Ionone	1MAI	206.2	191.1	191.1	161.2
PCMs	33704-61-9	Cashmeran	DPMI	206.2	191.1	191.0	91
PCMs	54464-57-2	Iso E super	OTNE	191.0	121	119.0	91
PCMs	13171-00-1	Celestolide	ADBI	229.2	173.1	244.2	229.2
PCMs	15323-35-0	Phantolide	AHMI	244.2	229.1	229.2	171.2
PCMs	68140-48-7	Traseolide	ATII	215.1	173.1	258.2	215.1
PCMs	1222-05-5	Galaxolide	HHCB	243.2	213.1	258.2	243.2
PCMs	21145-77-7	Tonalide	AHTN	258.2	243.2	243.1	159
MCMs	502-72-7	Exaltone	EXN	224.2	98.1	166.1	81
MCMs	541-91-3	Muskone	MUS	238.2	112.1	209.2	95.1
MCMs	106-02-5	Exaltolide	EXL	180.2	95.1	222.2	111.2
MCMs	7779-50-2	Ambrettolide	AMB	252.2	123.1	234.2	93
MCMs	109-29-5	16-Hexadecanolide	16-H	254.2	99.1	236.2	95
MCMs	54982-83-1	Musk MC-4	MC-4	213.1	149.1	173.1	111.1
MCMs	6707-60-4	Cervolide	CER	182.1	122	181.0	135
MCMs	105-95-3	Ethylene brassylate	EtB	187.1	125.1	227.2	163.1

Table S2(b). Multiple Reaction Monitoring (MRM) transitions in electron capture negative ion (ECNI) for NMs.

Chemicals	Quantifier precursor ion	Quantifier product ion	Qualifier Precursor ion	Qualifier product ion
MA	268	253	253	221
MX	267	175	267	147
D_{15}-MX	282	186	282	234
MM	278	203	278	159
MT	266	236	266	46
MK	264	219	264	206

Table S3. Concentrations [Mean (SD; Min-Max), in pg/m^3] of synthetic musk compounds in on-site and off-site air of **Lagoon** during the **summer period**.

**Lagoon Summer 2017**	Total (GFF + PUF)		Gas Phase		Particle Phase	
**MCMs**	**On-site**	**Off-site**	**On-site**	**Off-site**	**On-site**	**Off-site**
AMB	ND	ND	ND	ND	ND	ND
CER	ND	ND	ND	ND	ND	ND
EtB	44 (5.1; 39-48)	21	44 (5.1; 39-48)	21	ND	ND
EXN	ND	ND	ND	ND	ND	ND
EXL	ND	ND	ND	ND	ND	ND
16-H	43 (8.5; 34-51)	60	43 (8.6; 34-51)	60	ND	ND
MC-4	19 (16; ND-30)	13	19 (16; ND-30)	13	ND	ND
MUS	ND	ND	ND	ND	ND	ND
**NMs**						
MA	ND	ND	ND	ND	ND	ND
MK	32 (29; ND-53)	ND	33 (29; ND-53)	ND	ND	ND
MM	ND	ND	ND	ND	ND	ND
MT	ND	ND	ND	ND	ND	ND
MX	ND	ND	ND	ND	ND	ND
**PCMs**						
DPMI	25 (43; ND-75)	ND	25 (43; ND-75)	ND	ND	ND
ADBI	15 (13; 4.5-30)	ND	15 (13; 4.5-30)	ND	ND	ND
HHCB	8800 (11000; 830-21000)	200	8800 (11000; 830-21000)	200	3.5 (3.9; 0.80-7.9)	ND
OTNE	6200 (7400; 780-15000)	ND	6200 (7400; 780-15000)	ND	ND	ND
1MAI	21 (36; ND-62)	ND	21 (36; ND-62)	ND	ND	ND
AHMI	2.8 (4.8; ND-8.3)	ND	2.8 (4.8; ND-8.3)	ND	ND	ND
AHTN	720 (1000; ND-1900)	12	720 (1000; ND-1900)	12	ND	ND
ATII	ND	ND	ND	ND	ND	ND

ND: Not detected.

Table S4. Concentrations [Mean (SD; Min-Max), in pg/m^3] of synthetic musk compounds in on-site and off-site air of **Oxidation Ditch** during the **summer period**.

**Oxidation Ditch Summer 2017**	Total (GFF + PUF)		Gas Phase		Particle Phase	
**MCMs**	**On-site**	**Off-site**	**On-site**	**Off-site**	**On-site**	**Off-site**
AMB	ND	ND	ND	ND	ND	ND
CER	ND	ND	ND	ND	ND	ND
EtB	59 (3.2; 56-62)	20	59 (3.2; 56-62)	20	ND	ND
EXN	ND	ND	ND	ND	ND	ND
EXL	ND	ND	ND	ND	ND	ND
16-H	23(40; ND-69)	85	23 (40; ND-69)	85	ND	ND
MC-4	38 (2.4; 35-40)	12	38 (2.4; 35-40)	12	ND	ND
MUS	ND	ND	ND	ND	ND	ND
**NMs**						
MA	ND	ND	ND	ND	ND	ND
MK	44 (38; ND-68)	19	44 (38; ND-68)	19	ND	ND
MM	ND	ND	ND	ND	ND	ND
MT	ND	ND	ND	ND	ND	ND
MX	94(160; ND-280)	ND	94 (160; ND-280)	ND	ND	ND
**PCMs**						
DPMI	43 (43, 13-93)	ND	43 (43; 13-93)	ND	ND	ND
ADBI	15 (7.5; 11-24)	4.2	15 (7.5; 11-24)	ND	ND	ND
HHCB	4700 (2100; 3200-7000)	290	4700 (2100; 3200-7000)	280	6.0 (4.6; 0.90-10)	6.3
OTNE	4100 (1300; 3400-5600)	24	4100 (1300; 3400-5600)	ND	ND	ND
1MAI	79 (49; 37-130)	ND	79 (49; 37-130)	ND	ND	ND
AHMI	0.74 (1.3; ND-2.2)	ND	0.74 (1.3; ND-2.2)	ND	ND	ND
AHTN	440 (250; 270-730)	32	440 (250; 270-730)	32	ND	ND
ATII	ND	ND	ND	ND	ND	ND

ND: Not detected.

Table S5. Concentrations [Mean (SD; Min-Max), in pg/m^3] of synthetic musk compounds in on-site and off-site air of **CAS 1** during the **summer period**.

**CAS 1 Summer 2017**	Total (GFF + PUF)		Gas Phase Mean		Particle Phase	
**MCMs**	**On-site**	**Off-site**	**On-site**	**Off-site**	**On-site**	**Off-site**
AMB	ND	ND	ND	ND	ND	ND
CER	ND	ND	ND	ND	ND	ND
EtB	66 (4.6; 60-69)	27	65 (4.6; 60-69)	27	ND	ND
EXN	270 (160; 160-450)	ND	270 (160; 160-450)	ND	ND	ND
EXL	ND	ND	ND	ND	ND	ND
16-H	89 (30; 71-120)	67	89 (30; 71-120)	67	ND	ND
MC-4	28 (2.0; 26-30)	11	28 (2.0; 26-30)	11	ND	ND
MUS	ND	ND	41 (15; 31-53)	ND	ND	ND
**NMs**						
MA	ND	ND	ND	ND	ND	ND
MK	62 (6.4; 58-70)	ND	62 (6.4; 58-69)	ND	ND	ND
MM	28 (27; ND-53)	ND	28 (27; ND-53	ND	ND	ND
MT	ND	ND	ND	ND	ND	ND
MX	210 (15; 190-220)	ND	210 (15; 190-220)	ND	ND	ND
**PCMs**						
DPMI	910 (530; 460-1500)	32	910 (530; 460-1500)	32	ND	ND
ADBI	500 (300; 260-830)	7.3	480 (300; 250-820)	7.3	11 (0.65; 10-12)	ND
HHCB	460000 (240000; 260000-730000)	640	460000 (240000; 260000-730000)	640	200 (110; 74-280)	6.6
OTNE	650000 (410000; 290000-1100000)	17	650000 (410000; 300000-1100000)	17	320 (220; 110-550)	ND
1MAI	ND	ND	ND	ND	ND	ND
AHMI	160 (92; 83-260)	ND	160 (92; 83-260)	ND	ND	ND
AHTN	8200 (1600; 6500-9500)	69	8200 (1600; 6500-9500)	69	ND	ND
ATII	ND	ND	ND	ND	ND	ND

ND: Not detected.

Table S6. Concentrations [Mean (SD; Min-Max), in pg/m^3] of synthetic musk compounds in on-site and off-site air of **CAS 2** during the **summer period**.

CAS 2 Summer 2017	Total (GFF + PUF)		Gas Phase		Particle Phase	
**MCMs**	**On-site**	**Off-site**	**On-site**	**Off-site**	**On-site**	**Off-site**
AMB	ND	ND	ND	ND	ND	ND
CER	ND	ND	ND	ND	ND	ND
EtB	81 (5.4; 75-86)	27	81 (5.4; 75-86)	27	ND	ND
EXN	230 (18; 210-240)	ND	230 (18; 210-240)	ND	ND	ND
EXL	ND	ND	ND	ND	ND	ND
16-H	73 (24; 47-94)	55	73 (24; 47-94)	55	ND	ND
MC-4	35 (2.3; 32-36)	11	35 (2.3; 32-36)	11	ND	ND
MUS	ND	ND	ND	ND	ND	ND
**NMs**						
MA	ND	ND	ND	ND	ND	ND
MK	80 (5.0; 74-84)	18	80 (5.0; 74-84)	18	ND	ND
MM	ND	ND	ND	ND	ND	ND
MT	ND	ND	ND	ND	ND	ND
MX	260 (20; 240-270)	ND	260 (20; 240-270)	ND	ND	ND
**PCMs**						
DPMI	1300 (310; 1000-1600)	41	1300 (310; 1000-1600)	41	ND	ND
ADBI	520 (52; 460-570)	8.0	510 (53; 450-550)	8.0	14 (0.90; 13-14)	ND
HHCB	680000 (29000; 650000-700000)	2600	680000 (29000; 650000-700000)	2600	280 (120; 210-420)	4.4
OTNE	530000 (59000; 470000-590000)	2400	530000 (59000; 470000-590000)	2400	78 (32; 56-110)	ND
1MAI	ND	43	ND	43	ND	ND
AHMI	190 (24; 170-210)	3.6	190 (24; 170-210)	3.6	ND	ND
AHTN	11000 (1400; 9600-12000)	280	11000 (1500; 9600-12000)	280	9.3 (16; ND-28)	ND
ATII	ND	ND	ND	ND	ND	ND

ND: Not detected.

Table S7. Concentrations [Mean (SD; Min-Max), in pg/m^3] of synthetic musk compounds in on-site and off-site air of **Lagoon** during the **winter** period.

**Lagoon Winter 2017**	Total (GFF + PUF)		Gas Phase Mean		Particle Phase	
**MCMs**	**On-site**	**Off-site**	**On-site**	**Off-site**	**On-site**	**Off-site**
AMB	ND	ND	ND	ND	ND	ND
CER	ND	ND	ND	ND	ND	ND
EtB	140 (70; 60-190)	ND	69 (6.9; 61-73)	ND	73 (63; ND-120)	ND
EXN	ND	ND	ND	ND	ND	ND
EXL	ND	ND	ND	ND	ND	ND
16-H	26 (45; ND-77)	ND	26 (45; ND-77)	ND	ND	ND
MC-4	37 (32; ND-56)	ND	37 (32; ND-56)	ND	ND	ND
MUS	ND	ND	ND	ND	ND	ND
**NMs**						
MA	ND	ND	ND	ND	ND	ND
MK	36 (62; ND-110)	ND	36 (62; ND-110)	ND	ND	ND
MM	ND	ND	ND	ND	ND	ND
MT	ND	ND	ND	ND	ND	ND
MX	ND	ND	ND	ND	ND	ND
**PCMs**						
DPMI	110 (110; ND-230)	ND	83 (84; ND-170)	ND	20 (34; ND-58)	ND
ADBI	84 (64; 47-160)	ND	52 (9.4; 47-64)	ND	31 (54; ND-94)	ND
HHCB	1400 (200; 1200-1500)	ND	1300 (170; 1200-1500)	ND	91 (160; ND-270)	ND
OTNE	1600 (300; 1400-2000)	ND	1600 (220; 1400-1800)	ND	46 (80; ND-140)	ND
1MAI	132; (230; ND-400)	ND	130 (230; ND-400)	ND	ND	ND
AHMI	100 (59; 65-170)	ND	67 (1.7; 65-69)	ND	33 (57; ND-99)	ND
AHTN	410 (320; 200-770)	ND	380 (260; 200-680)	ND	34 (59; ND-100)	ND
ATII	ND	ND	ND	ND	ND	ND

ND: Not detected.

Table S8. Concentrations [Mean (SD; Min-Max), in pg/m^3] of synthetic musk compounds in on-site and off-site air of **Oxidation Ditch** during the **winter** period.

**Oxidation Ditch Winter 2017**	Total (GFF + PUF)		Gas Phase		Particle Phase	
**MCMs**	**On-site**	**Off-site**	**On-site**	**Off-site**	**On-site**	**Off-site**
AMB	ND	ND	ND	ND	ND	ND
CER	ND	ND	ND	ND	ND	ND
EtB	60 (12; 46-69)	350	60 (12; 46-69)	350	ND	210
EXN	ND	ND	ND	ND	ND	ND
EXL	ND	ND	ND	ND	ND	ND
16-H	45 (45; ND-90)	140	45 (45; ND-90)	140	ND	ND
MC-4	28 (24; ND-43)	84	28 (24; ND-43)	84	ND	ND
MUS	ND	ND	ND	ND	ND	ND
**NMs**						
MA	ND	ND	ND	ND	ND	ND
MK	ND	ND	ND	ND	ND	ND
MM	ND	ND	ND	ND	ND	ND
MT	ND	ND	ND	ND	ND	ND
MX	ND	ND	ND	ND	ND	ND
**PCMs**						
DPMI	28 (25; ND-47)	ND	28 (25; ND-47)	ND	ND	ND
ADBI	120 (14; 100-130)	ND	50 (3.7; 46-53)	ND	67 (16; 49-78)	ND
HHCB	1300 (170; 1200-1500)	280	1300 (170; 1200-1500)	280	31 (4.2; 28-35)	3.0
OTNE	1600 (220; 1400-1800)	ND	1600 (220; 1400-1800)	ND	1.3 (2.2; ND-3.9)	ND
1MAI	ND	ND	ND	ND	ND	ND
AHMI	54 (4.7; 50-60)	ND	55 (4.7; 50-60)	ND	67 (16; 49-78)	ND
AHTN	300 (160; 110-400)	160	260 (130; 110-350)	160	41 (36; ND-68)	ND
ATII	ND	ND	ND	ND	ND	ND

ND: Not detected.

Table S9. Concentrations [Mean (SD; Min-Max), in pg/m^3] of synthetic musk compounds in on-site and off-site air of **CAS 1** during the **winter** period.

**CAS 1 Winter 2017**	Total (GFF + PUF)		Gas Phase		Particle Phase	
**MCMs**	**On-site**	**Off-site**	**On-site**	**Off-site**	**On-site**	**Off-site**
AMB	ND	ND	ND	ND	ND	ND
CER	ND	ND	ND	ND	ND	ND
EtB	110 (3.8; 110-120)	320	44 (2.1; 42-46)	320	70 (2.2; 67-72)	ND
EXN	ND	ND	ND	ND	ND	ND
EXL	ND	ND	ND	ND	ND	ND
16-H	35 (34; ND-67)	ND	35 (34; ND-67)	ND	ND	ND
MC-4	33 (1.9; 32-35)	100	33 (1.9; 32-35)	100	ND	ND
MUS	ND	ND	ND	ND	ND	ND
**NMs**						
MA	ND	ND	ND	ND	ND	ND
MK	80 (86; ND-170)	ND	49 (42; ND-76)	ND	31 (54; ND-94)	ND
MM	ND	ND	ND	ND	ND	ND
MT	ND	ND	ND	ND	ND	ND
MX	93 (2.7; 91-96)	ND	93 (2.7; 91-96)	ND	ND	ND
**PCMs**						
DPMI	850 (180; 700-1100)	ND	820 (170; 680-1000)	ND	30 (8.8; 20-35)	ND
ADBI	290 (2.4; 290)	80	230 (7.1; 230-240)	80	55 (6.3; 48-59)	ND
HHCB	110000 (21000; 95000-130000)	270	100000 (9000; 95000-110000)	270	8200 (12000; 490-22000)	ND
OTNE	360000 (68000; 290000-410000)	720	360000 (68000; 290000-410000)	720	780 (640; 140-1400)	ND
1MAI	ND	ND	ND	ND	ND	ND
AHMI	170 (4.5; 160-170)	ND	110 (3.9; 100-110)	ND	60 (1.2; 58-61)	ND
AHTN	4500 (480; 3900-4900)	180	4200 (570; 3700-4800)	180	240 (120; 96-320)	ND
ATII	ND	ND	ND	ND	ND	ND

ND: Not detected.

Table S10. Concentrations [Mean (SD; Min-Max), in pg/m^3] of synthetic musk compounds in on-site and off-site air of **CAS2** during the **winter** period.

**CAS2 Winter 2017**	Total (GFF + PUF)		Gas Phase		Particle Phase	
**MCMs**	**On-site**	**Off-site**	**On-site**	**Off-site**	**On-site**	**Off-site**
AMB	ND	ND	ND	ND	ND	ND
CER	ND	ND	ND	ND	ND	ND
EtB	110 (4.7; 100-110)	39	35 (2.0; 34-38)	39	70 (2.7; 67-72)	ND
EXN	ND	ND	ND	ND	ND	ND
EXL	ND	ND	ND	ND	ND	ND
16-H	47 (9.1; 38-56)	25	47 (9.1; 38-56)	25	ND	ND
MC-4	31 (3.9; 26-34)	12	31 (4.0; 26-34)	12	ND	ND
MUS	ND	ND	ND	ND	ND	ND
**NMs**						
MA	ND	ND	ND	ND	ND	ND
MK	96 (48; 65-150)	22	66 (4.4; 63-72)	22	29 (51; ND-88)	ND
MM	ND	ND	ND	ND	ND	ND
MT	ND	ND	ND	ND	ND	ND
MX	95 (4.6; 92-100)	ND	95 (4.6; 92-100)	ND	ND	ND
**PCMs**						
DPMI	730 (140; 580-840)	ND	700 (140; 550-810)	ND	36 (0.84; 35-36)	ND
ADBI	310 (37; 290-350)	32		32	61 (3.8; 58-65)	ND
HHCB	100000 (16000; 86000-120000)	510	100000 (20000; 78000-120000)	500	ND	10
OTNE	330000 (63000; 260000-380000)	1300	330000 (65000; 260000-380000)	1300	1300 (1300; 460-2900)	ND
1MAI	1000 (940; ND-1800)	120	1000 (940; ND-1800)	120	ND	ND
AHMI	170 (10; 160-180)	14	110 (12; 100-120)	14	61 (3.8; 58-65)	ND
AHTN	5100 (690; 4600-5900)	130	4500 (920; 3700-5500)	130	530 (510; 120-1100)	ND
ATII	ND	ND	ND	ND	ND	ND

ND: Not detected.

Table S11. Concentrations (Mean ± SD, ng/L) of SMCs in influent and effluent samples from four wastewater treatment plants during the winter season.

	CAS1-Influent	CAS1-Effluent	CAS2-Influent	CAS2-Effluent	Oxidation Ditch-Influent	Oxidation Ditch-Effluent	Lagoon-Influent	Lagoon-Effluent
	PCMs							
DPMI	120 ±65	36±14	79 ±12	55 ±6.9	12 ±5.1	7.1 ±2.0	170 ±75	3.4 ±1.5
1MAI	1200 ±570	17±10	1200 ±150	5.5 ±3.0	220±130	ND	1900±400	2.4 ±0.0
OTNE	15400 ±6450	3800±660	12000 ±1100	4600 ±500	2700±870	620 ±65	12700±3210	26 ±12
ADBI	38 ±22	14 ±3.2	39 ±5.7	16 ±2.9	18 ±6.3	5.5 ±0.44	33 ±5.7	ND
ATII	40 ±20	10 ±1.9	31 ±11	14 ±3.8	13 ±3.8	1.6 ±0.55	38 ±19	1.0 ±1.1
HHCB	14200 ±3980	4600 ±410	13300 ±1360	5800 ±340	6100 ±3800	2200 ±190	13600 ±2700	210 ±33
AHTN	680 ±190	170 ±37	610 ±83	230 ±43	400 ±200	97 ±13	600 ±240	6.9 ±3.0
	MCMs							
EtB	18±7.1	4.7 ±2.2	6.9±2.0	2.8 ±2.1	7.1±3.9	1.2 ±0.59	110±82	4.3 ±2.4
	NMs							
MA	1.5	ND	ND	ND	ND	ND	ND	ND
MK	37	11	19	19	17	5.1	5.7	ND
MM	1.5	ND	0.97	ND	1.3	ND	ND	ND
MT	1.6	ND	ND	ND	0.97	ND	ND	ND
MX	8.7	7.1	3.2	3.1	12	5.9	ND	ND

ND: Not detected.

Table S12. Chemical properties [Log K_{OW}, vapour pressure (VP), water solubility (WS), Henry's low constant (HLC), Log K_{AW}, and Log K_{OA}] for SMCs.

Chemical	Log K_{OW} [a]	VP (Pa) [a]	WS (mg/L) [a]	HLC (Pa m^3)/mol [b,c]	Log K_{AW} [a]	Log K_{OA} [a]
MT	5.18	0.00052	0.2946	7.3E+01	-4.925	10.105
MA	4.17	0.00175	2.107	2.0E+01	-6.239	10.409
MM	5.39	0.00033	0.1661	1.9E+02	-5.077	10.467
MK	4.31	7.78E-05	1.148	7.2E+00	-5.602	9.902
MX	4.45	3.00E-05	0.3392	3.1E+01	-5.133	10.033
1MAI	4.78	0.868	3.328	2.4E+01	-2.008	6.788
DPMI	4.49	0.537	5.937	9.4E+00	-2.236	6.726
OTNE	5.18	0.147	1.077	4.7E+01	-1.716	6.896
ADBI	5.93	0.0192	0.2202	3.2E+00	-2.886	8.816
AHMI	5.85	0.0195	0.2545	6.1E+02	-2.886	8.736
ATII	6.31	0.00911	0.0869	7.9E+01	-2.763	9.073
HHCB	5.9	0.0683	0.1943	4.2E+01	-2.363	8.263
AHTN	5.7 [d]	0.0252	0.2879	1.4E+01	-2.249	7.949
EXN	5.55	0.0557	0.5989	6.5E+01	-1.572	7.122
MCN	5.96	0.0625	0.2213	8.6E+01	-1.449	7.409
EXL	6.15	0.00689	0.1484	2.3E+02	-1.023	6.15
AMB	5.37	0.00299	0.5925	2.7E+02	-0.957	6.327
16-H	6.65	0.0033	0.04727	3.1E+02	-0.90	7.55
MC-4	4.22	0.00033	5.417	2.3E-01	-4.016	8.236
CER	4.9	0.0021	1.433	2.0E+00	-3.079	7.979
EtB	4.71	5.85E-05	1.719	3.1E-01	-3.893	8.603

[a] Chemical properties estimated by US EPI SUITE V4.11[5]

[b] $K_{AW} = HLC/(R \times T)$

[c] Reference[4]

[d] Reference[8]

Table S13. The values to calculate the half-lives (HL) in air, water, and biota.

	BIOWIN 4 [a]	BIOWIN 3[a]	HL-Air (h) [a]	HL-water (h) [a]	Biodegradation (h) [a,b]
MT	2.89 (weeks)	1.8348 (months)	175	4320	2040
MA	3.10 (weeks)	1.9221 (months)	171	4320	2040
MM	2.85 (weeks)	1.7459 (recalcitrant)	146	4320	2112
MK	2.89 (weeks)	1.8253 (months)	199	4320	2040
MX	2.80 (weeks)	1.6717 (recalcitrant)	309	4320	2880
1MAI	3.3744 (days-weeks)	2.5086 (weeks-months)	0.466	900	358
DPMI	3.22 (weeks)	2.2965 (weeks-months)	2.16	900	358
OTNE	3.18 (weeks)	2.2345 (months)	0.469	1440	2040
ADBI	3.10 (weeks)	2.1376 (months)	34.5	1440	2040
AHMI	3.10 (weeks)	2.1376 (months)	16.9	1440	2040
ATII	3.16 (weeks)	2.2438 (months)	13.2	1440	2040
HHCB	3.09 (weeks)	2.1204 (months)	9.87	1440	2040
AHTN	3.08 (weeks)	2.1066 (months)	14.5	1440	2040
EXN	3.5018 (days-weeks)	2.6808 (weeks-months)	10.3	900	358
MCN	3.4815 (days-weeks)	2.6498 (weeks-months)	8.61	900	358
EXL	3.7299 (days-weeks)	2.8082 (weeks)	13.4	360	200
AMB	3.7125 (days-weeks)	2.7816 (weeks)	0.986	360	200
16-H	3.7096 (days-weeks)	2.7772 (weeks)	12.5	360	200
MC-4	3.9358 (days)	2.9131 (weeks)	16.4	360	200
CER	3.6971 (days-weeks)	2.7641 (weeks)	7.22	360	200
EtB	3.9156 (days)	2.8821 (weeks)	15	360	200

[a] Chemical properties estimated by US EPI SUITE V4.11[5]

[b] Reference[9]

Table S14. The environmental properties used for the model.

Parameter	Unit	Remark	Average Value
MTC_L	m/h	MTC: Mass Transfer Coefficient for Liquid	0.05
MTC_G	m/h	MTC: Mass Transfer Coefficient for gas	5.00
AeroDep	m/h	Aerosol Deposition Rate	7.20
T_{air}	K	Air Temperature	275.15
T_{water}	K	Water Temperature	286.15
WindSpeed	m/h	Windspeed	17280.00
AirFR	m^3/h	Air Flow Rate	9.9E+06
DensAero	kg/m^3	Density of Aerosol	1500
TSP	ug/m^3	Total suspended particulate	30.00
$C_{airOffSite}$	pg/m^3	Concentration in the off-site air	Measurement
C_{Inf} (4)	ng/L	Concentration in the influent (4)	Measurement
Number of Tanks		Primary (5) + Aeration (6) + Secondary (7)	3
Area (5)	m^2	Area (Primary Tank)	4592
Depth (5)	m	Depth (Primary Tank)	3.66
RemFlow	%	Remove Flow/water to sludge (Primary Tank)	0.24
Area (6)	m^2	Area (Aeration Tank)	3143
Depth (6)	m	Depth (Aeration Tank)	4.73
TSS Conc (6)	g/m^3	Outflow Total suspended solids (Aeration Tank)	1000
AerationRate (6)	m^3/h	Aeration Rate	25500
Area (7)	m^2	Area (Secondary Tank)	5403
Depth (7)	m	Depth (Secondary Tank)	3.60
Recycle Flow (7)	%	Recycle Flow (Secondary Tank)	38
Remove Flow (7)	%	Remove Flow/water to sludge (Secondary Tank)	1.1
Height of air	m	Height of air	5.00
FractRem (5)	na	Fraction of TSS Removed in the primary tank	0.30
Influent TSS Conc	g/m^3	Total suspended solids in the influent	275.00
InFR	m^3/h	Influent Flow Rate	3344.17

Note: The numbers in the brackets represent the compartments included in the fugacity model.

S3. Supplementary Figures

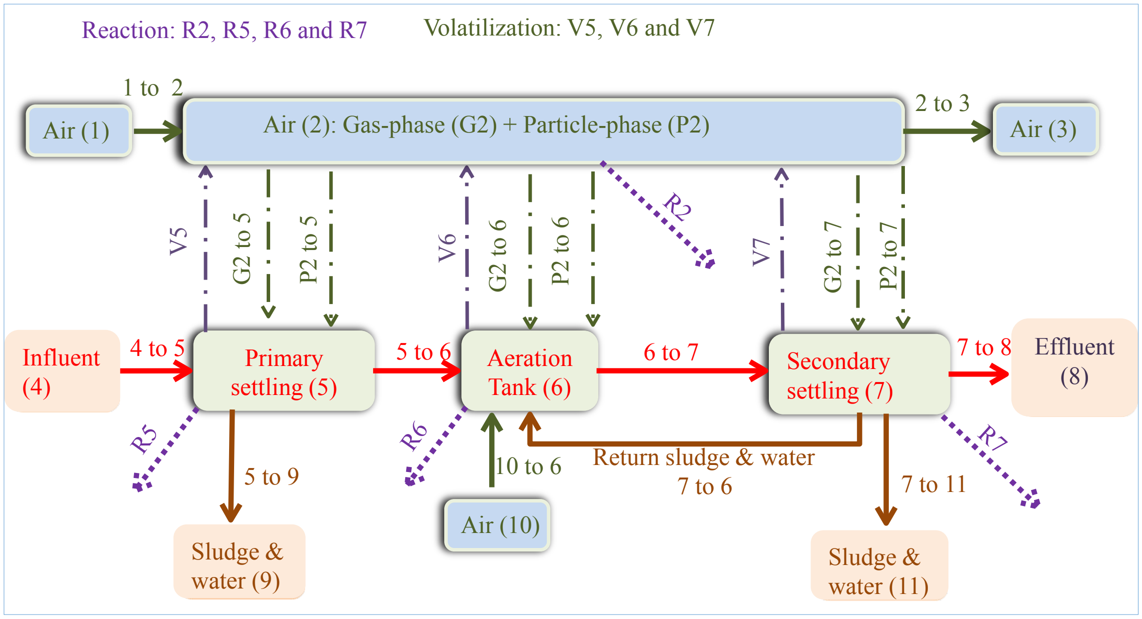

Figure S1. Diagram of fugacity transport and process in the studied WWTP.

Note: The numbers in the brackets represent the compartments included in the fugacity model.

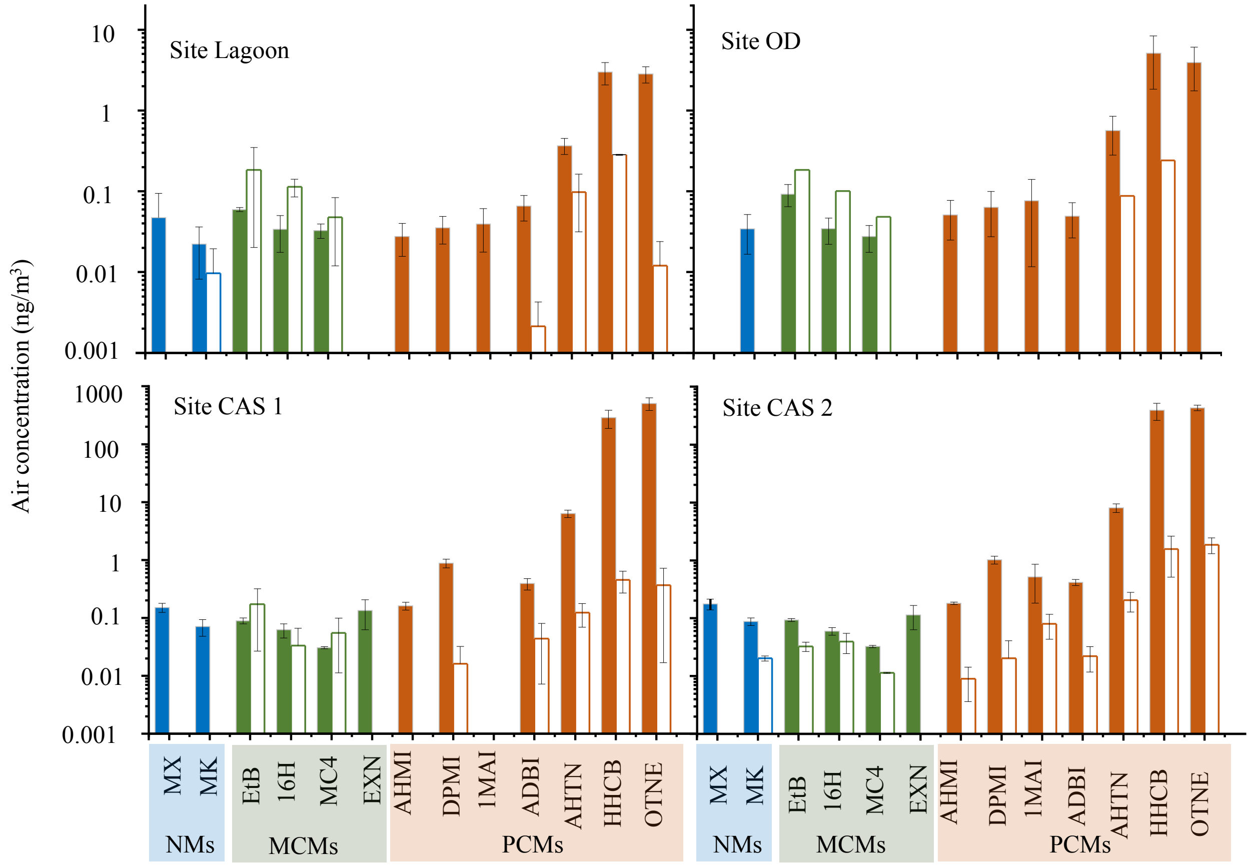

Figure S2. Average SMC concentrations (gas + particle) in the on-site (solid bars) and off-site (open bars) air samples collected at four WWTPs in Ontario, Canada in the winter and summer. Note that CAS represents conventional activated sludge, and OD represents oxidation ditch. When a bar is not shown, it indicates that the SMC was non-detectable in the sample.

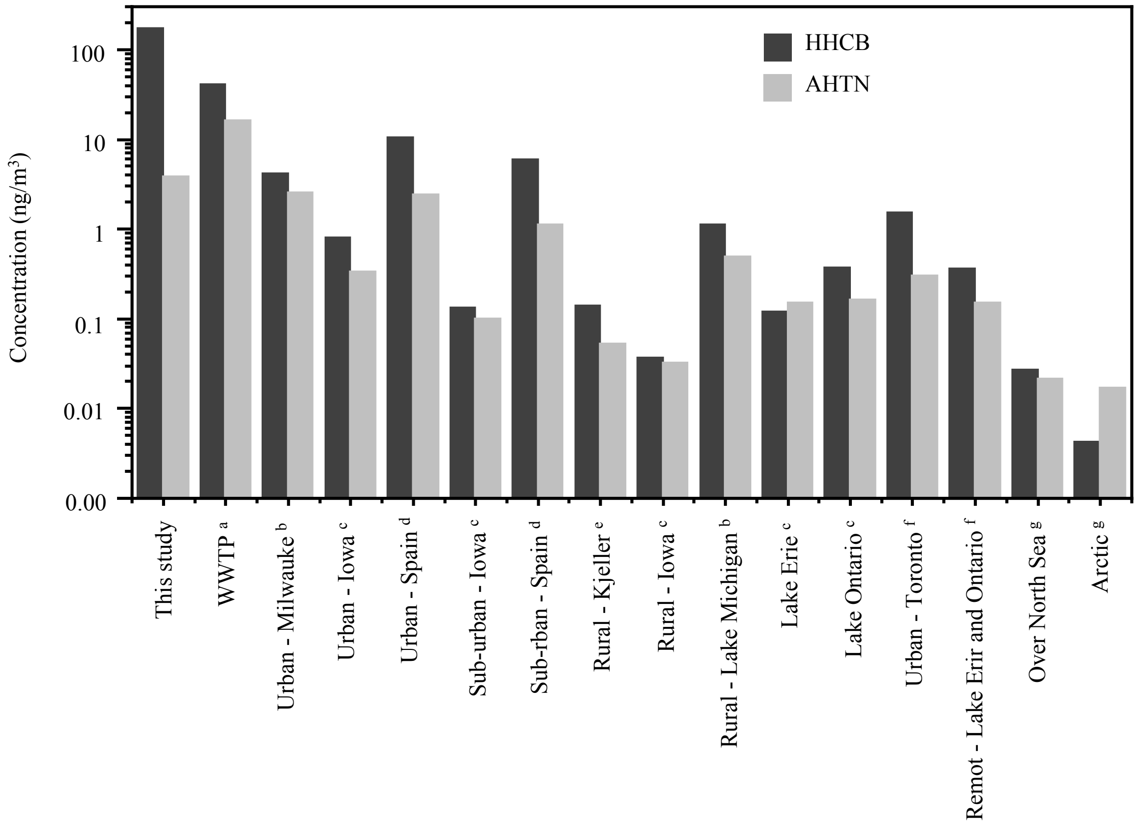

Figure S3. Comparisons of concentrations of HHCB and AHTN in air measured in this study with those reported in previous studies.

Note that the data were taken from [a]: Wong et al., 2019;[10] [b]: Peck and Hornbuckle, 2004;[8] [c]: Peck and Hornbuckle, 2006;[11] [d]: Ramirez et al., 2010;[12] [e]: Kallenborn et al., 1999;[13] [f]: McDonough et al., 2016;[14] [g]: Xie et al., 2007[15].

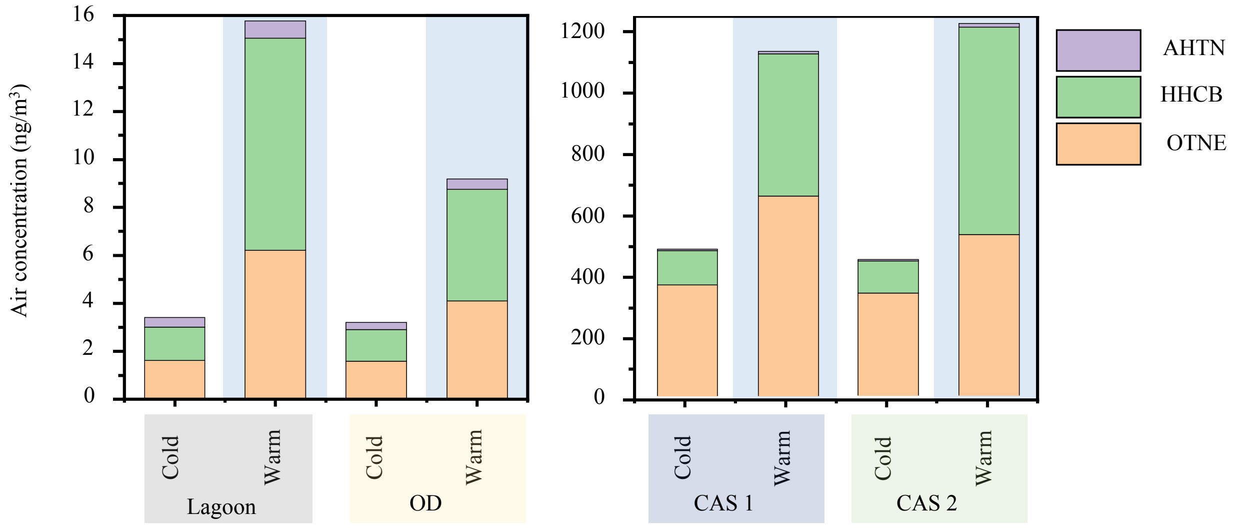

Figure S4. Comparisons of the concentrations of AHTN, HHCB, and OTNE in the air of WWTPs during warm and cold seasons.

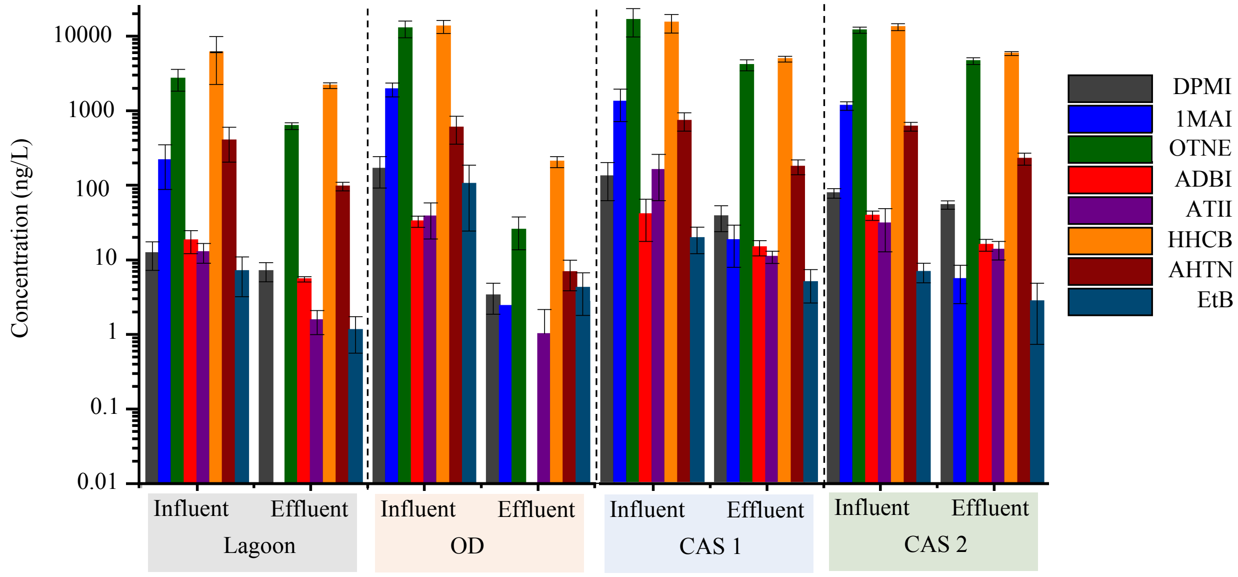

Figure S5. The concentrations of PCMs and MCMs in influent and effluent of four WWTPs (CAS: conventional activated sludge; OD: oxidation ditch) during the cold season.

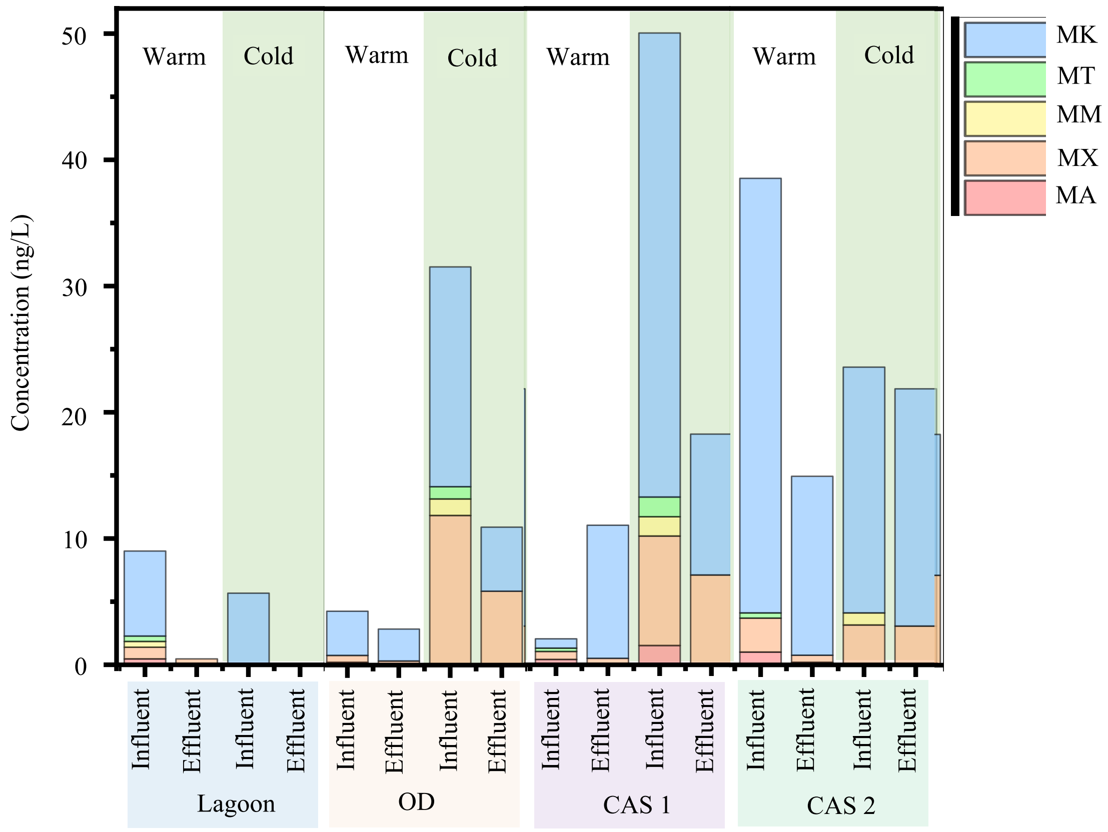

Figure S6. Concentrations of NMs in influent and effluent of four WWTPs (OD: oxidation ditch, CAS: conventional activated sludge) during warm and cold seasons.

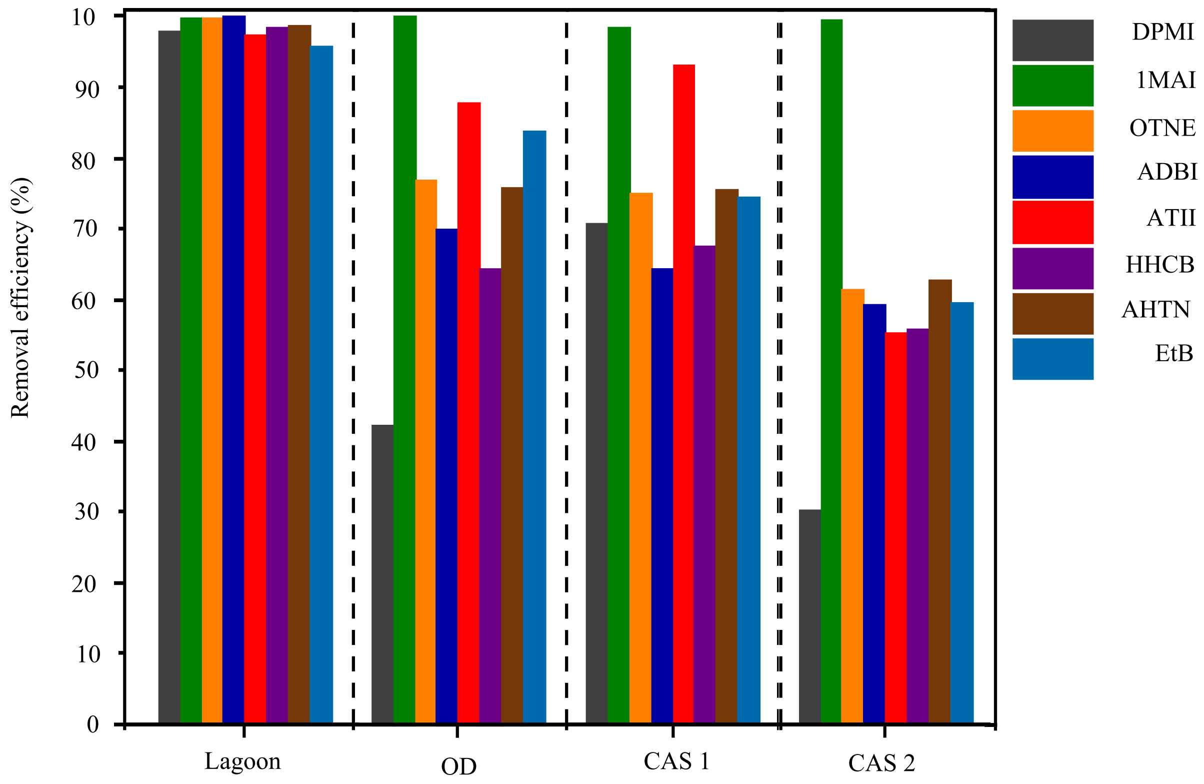

Figure S7. Removal efficiency for SMCs in four WWTPs during the cold season.

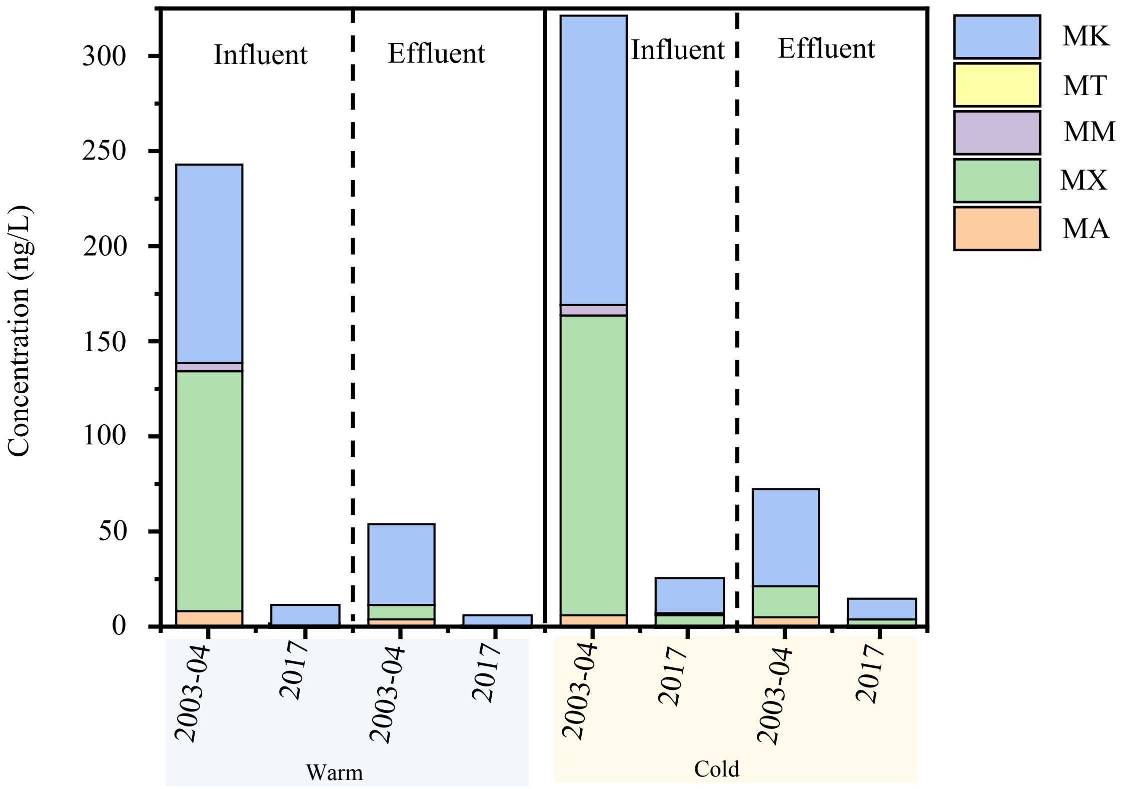

Figure S8. Comparisons between levels of NMs in influent and effluent of WWTP between 2003/2004 and 2017. Comparisons were performed for the warm season and cold season separately considering seasonal fluctuations in SMC concentrations.

Results from this study are presented as the average concentrations of NMs in four WWTPs. Both studies (this study and Smyth et al., 2008[16]) used the same extraction and clean-up methods but Smyth et al., 2008 used GC-MS to analyze NMs in the sample. Data derived from GC-MS and GC-MSMS were compared, and a good agreement was obtained between the two instrument methods.

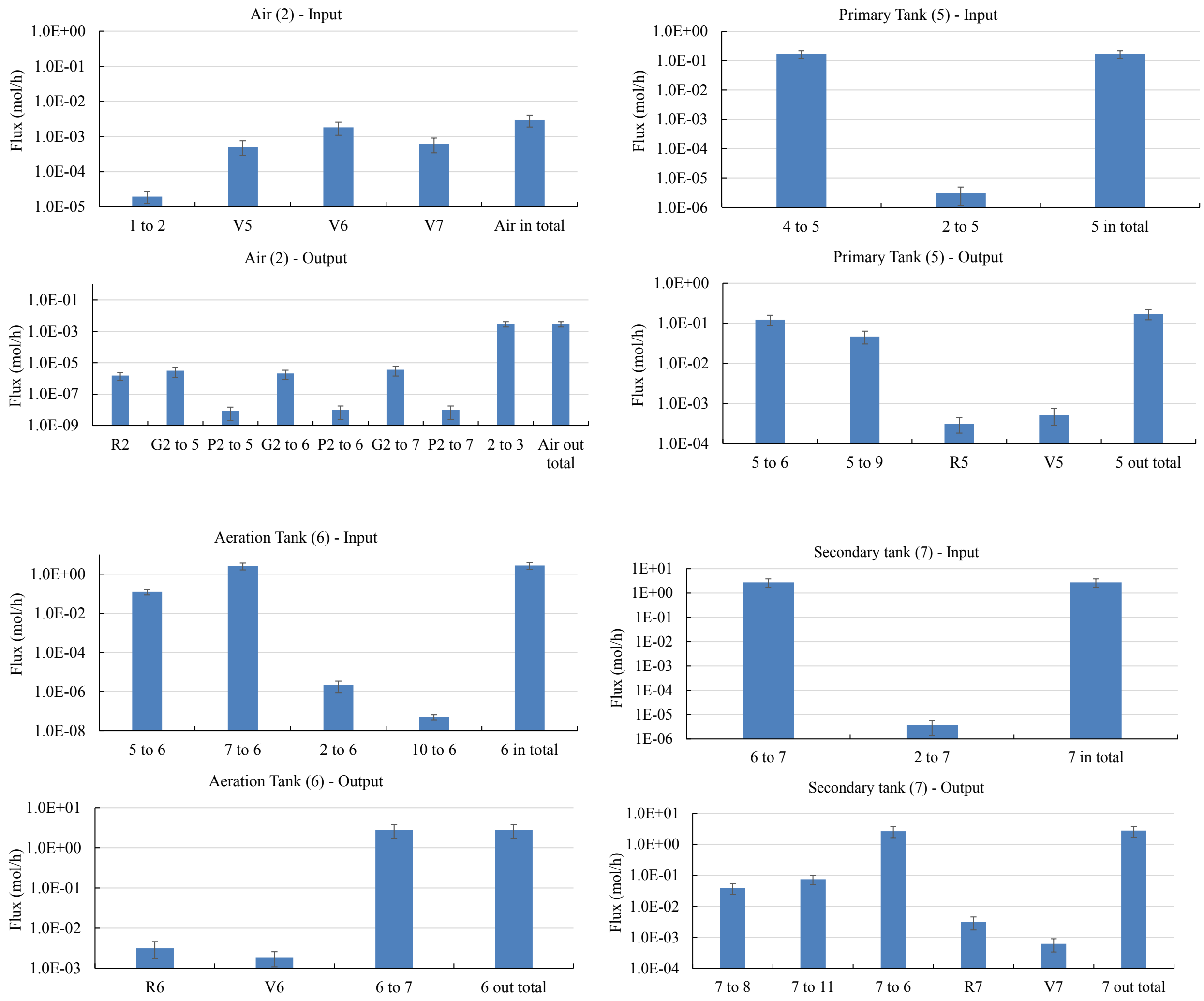

Figure S9. Mass Balance of HHCB in the CAS 2 WWTP in the winter season.

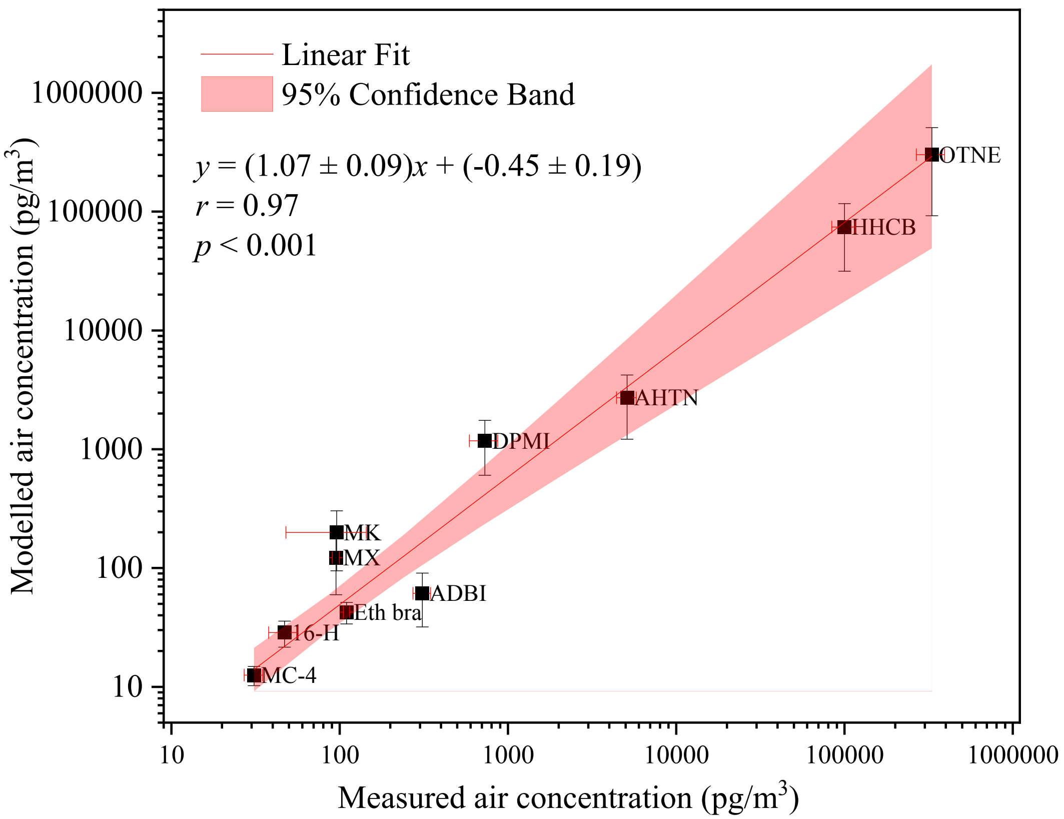

Figure S10. Correlations between measured air concentration and modeled air concentration.

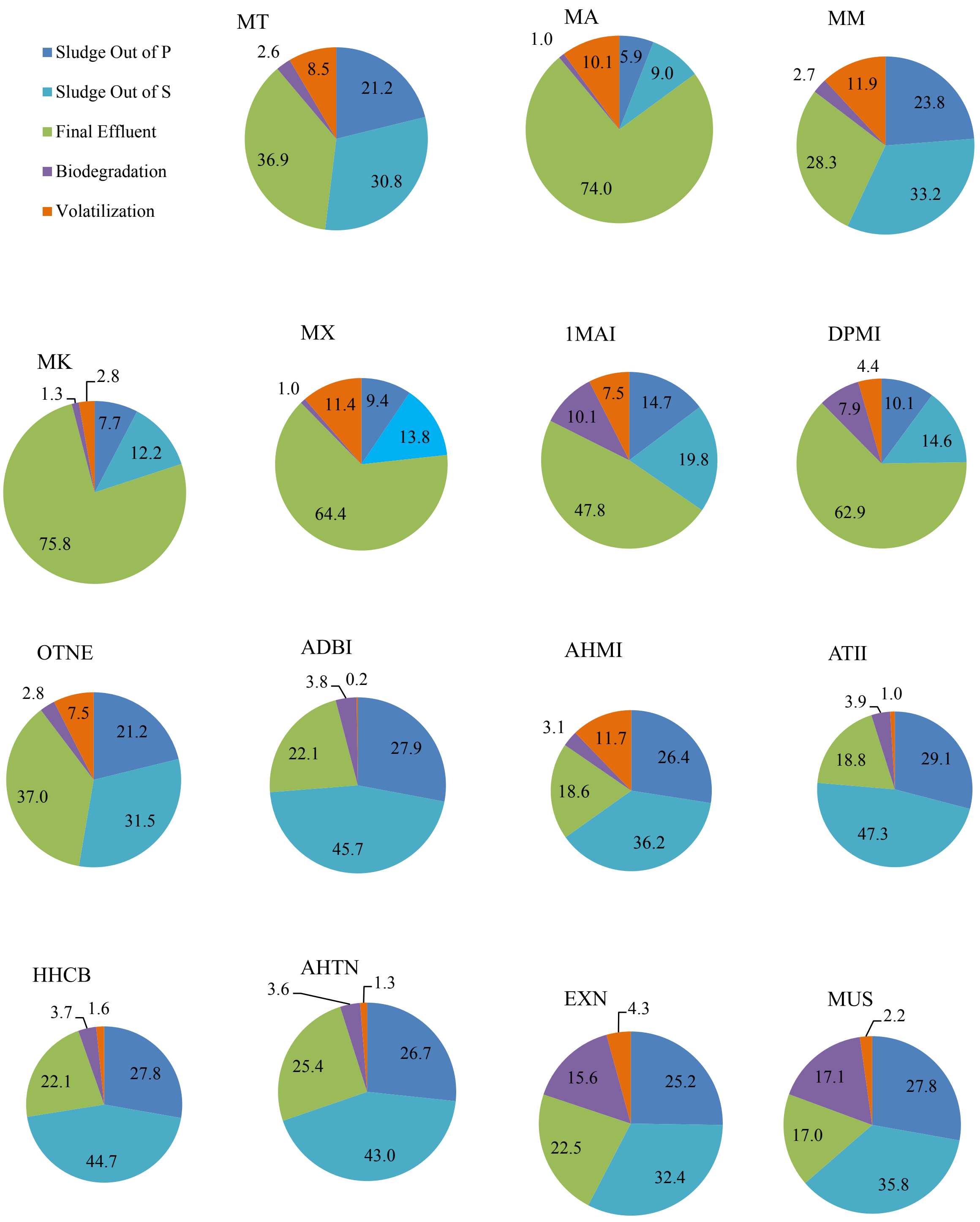

Figure S11. Estimated proportions (%) of advection, sorption, volatilization, and biotransformation of the studied SMCs in the WWTP.

Figure S11 (Continued)

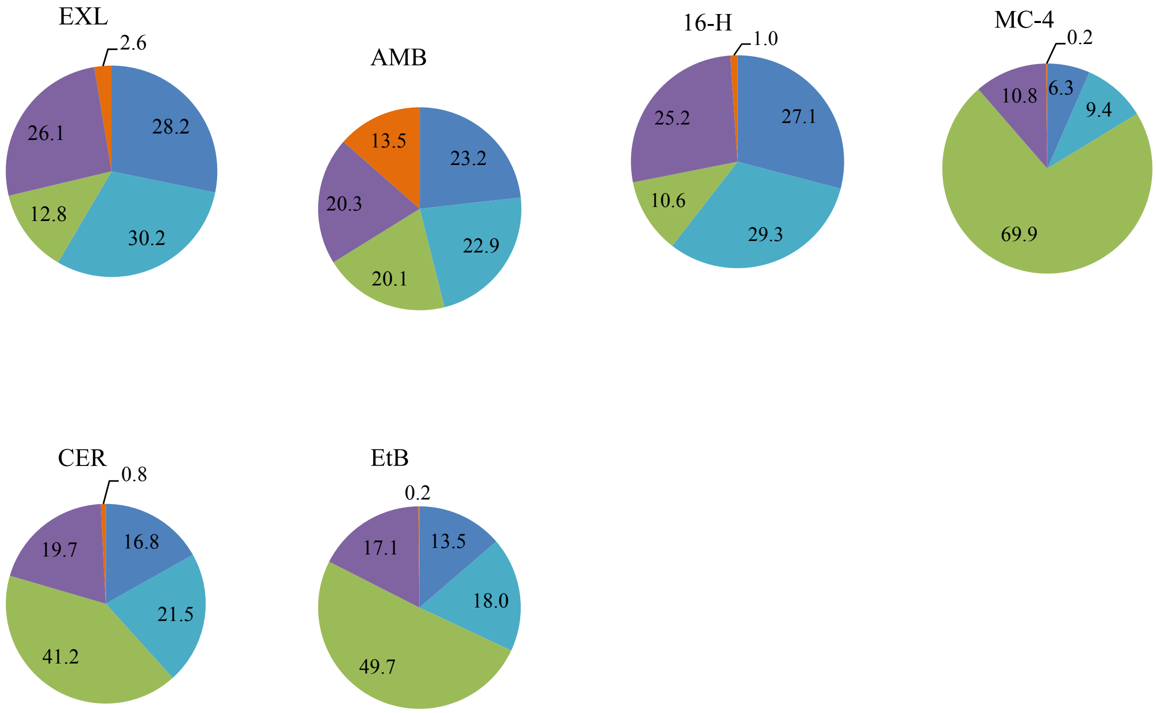

Figure S11 (Continued). Estimated proportions (%) of advection, sorption, volatilization, and biotransformation of SMCs in the WWTP.

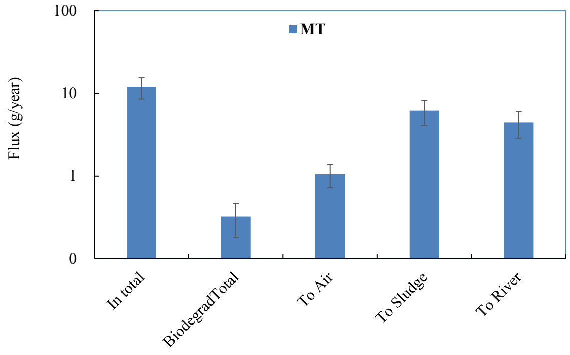

(a) MT

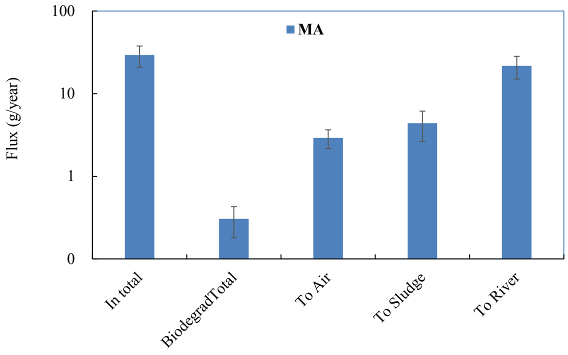

(b) MA

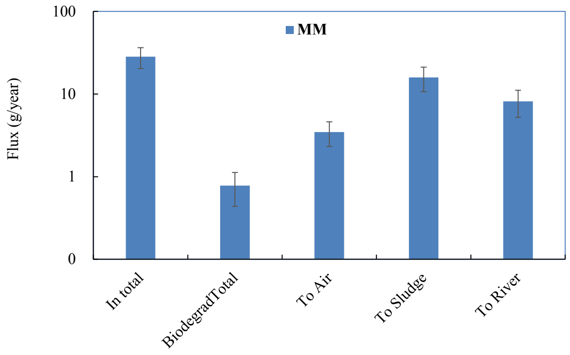

(c) MM

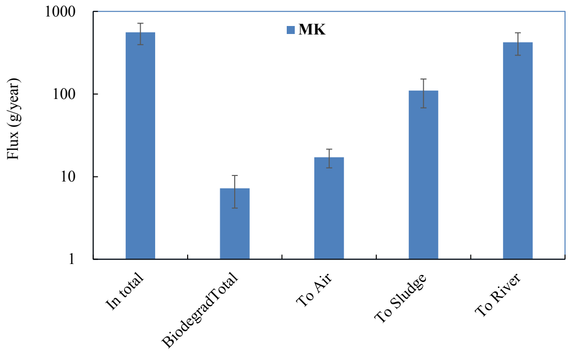

(d) MK

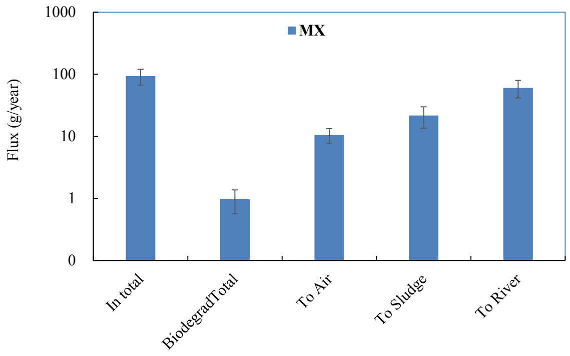

(e) MX

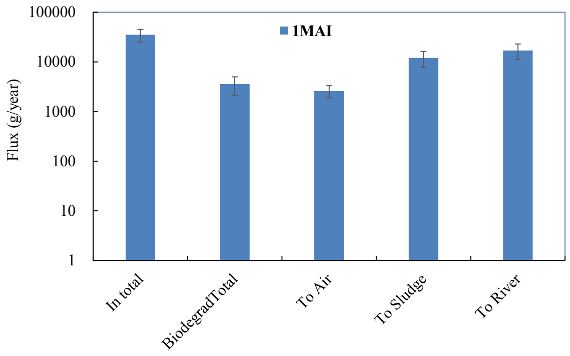

(f) 1MAI

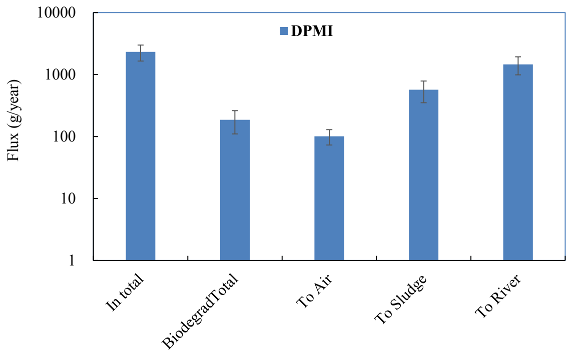

(g) DPMI

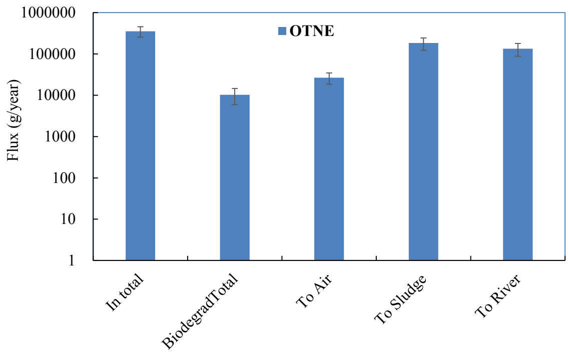

(h) OTNE

Figure S12. Contributions of HHCB from WWTPs to atmospheric and aquatic environments.

Figure S12 (Continued)

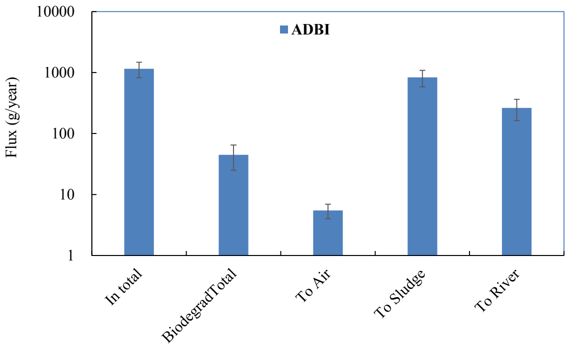

(i) ADBI

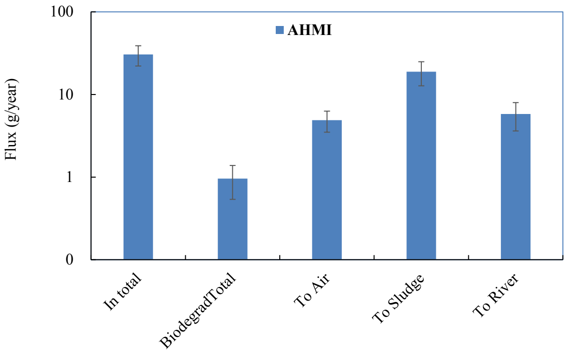

(j) AHMI

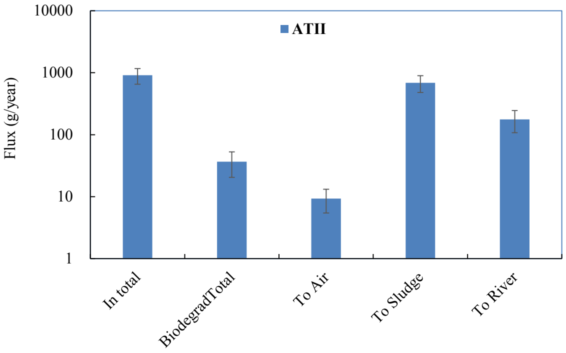

(k) ATII

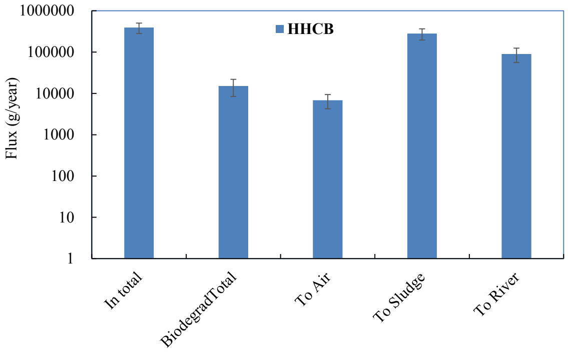

(l) HHCB

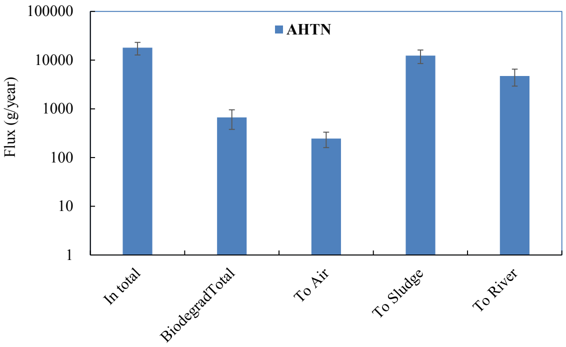

(m) AHTN

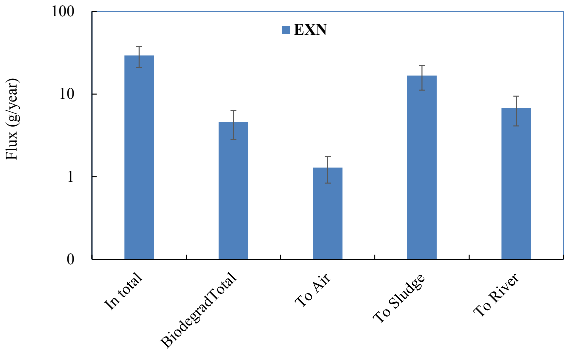

(n) EXN

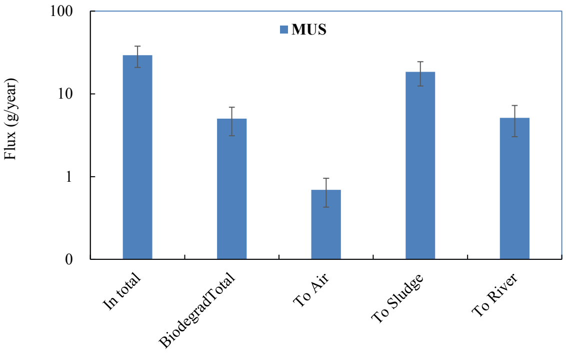

(o) MUS

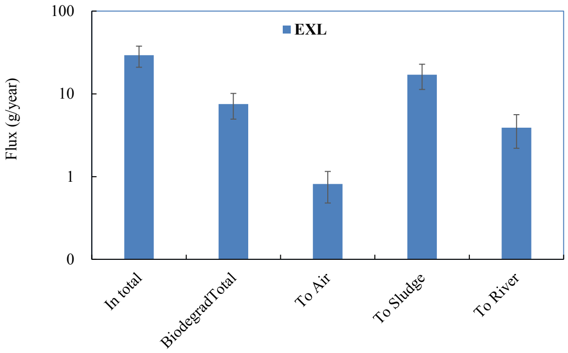

(p) EXL

Figure S12 (Continued)

Figure S12 (Continued)

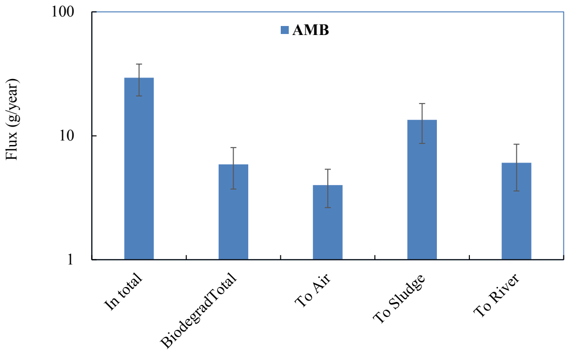

(q) AMB

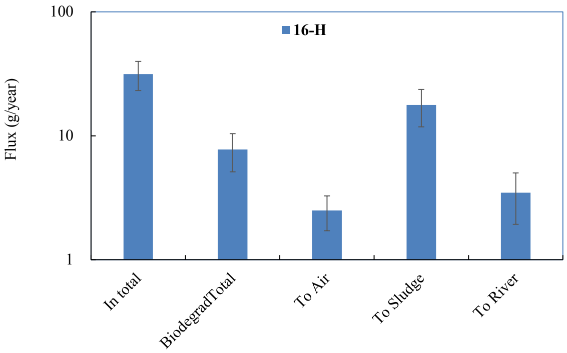

(r) 16-H

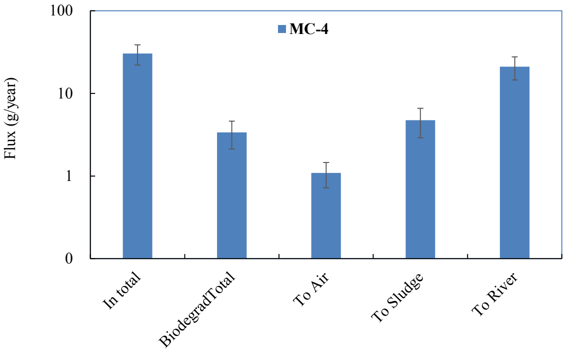

(s) MC-4

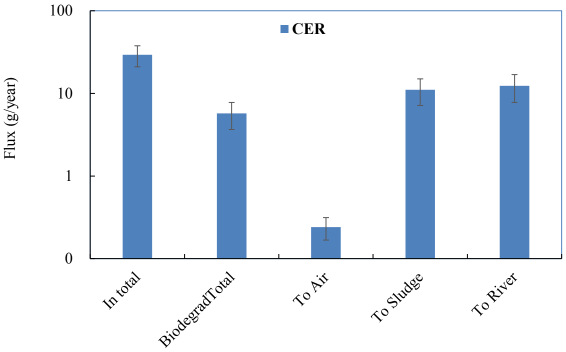

(t) CER

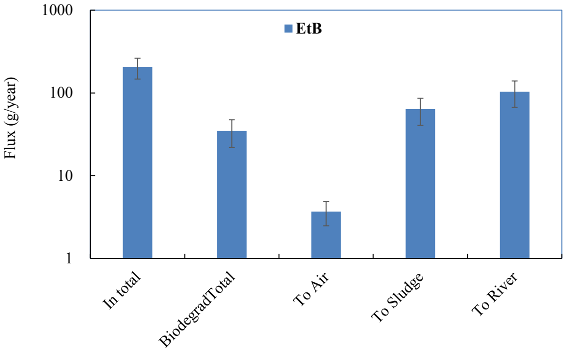

(u) EtB

Figure S12 (Continued). Contributions of SCMs from WWTPs to atmospheric and aquatic environments.

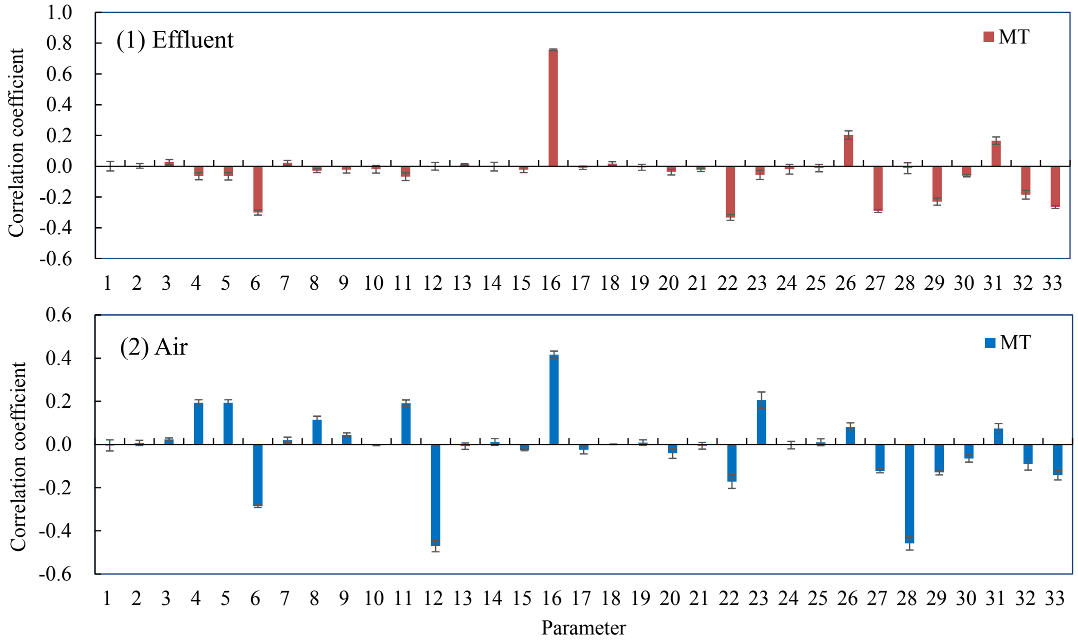

(a) MT

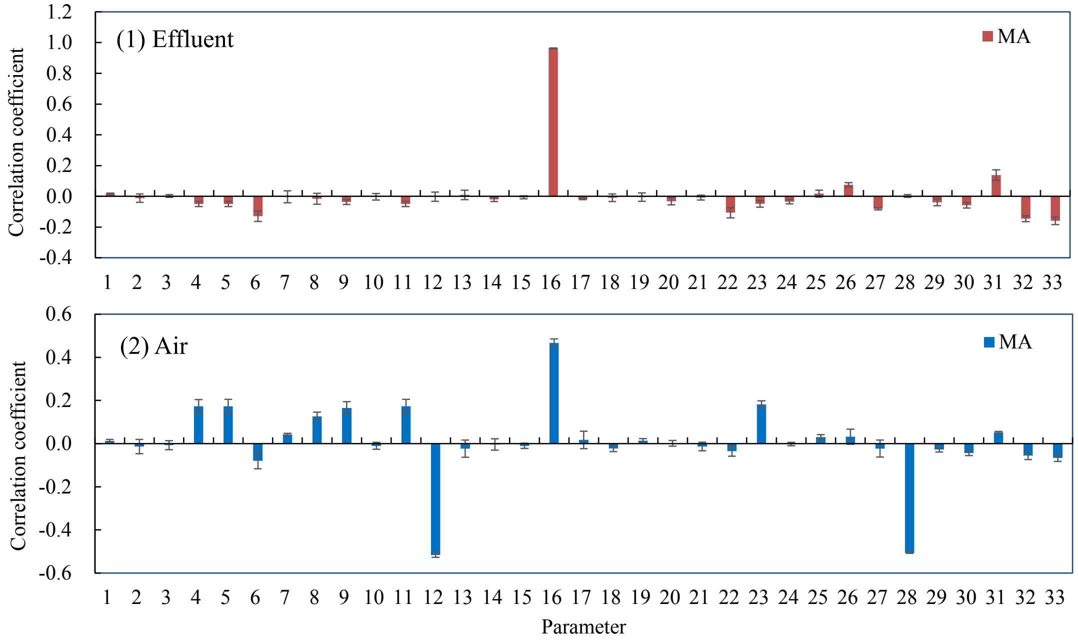

(b) MA

Figure S13. Sensitivity analysis of parameters using the correlation coefficient of each parameter and the output effluent and air concentrations for each SMC.

Figure 13 (Continue)

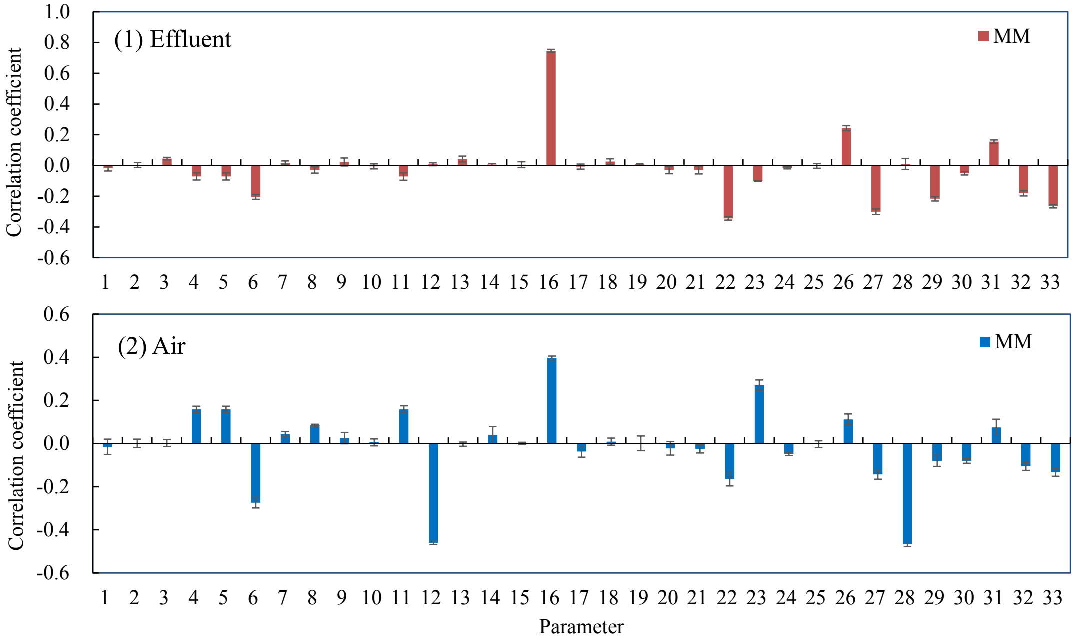

(c) MM

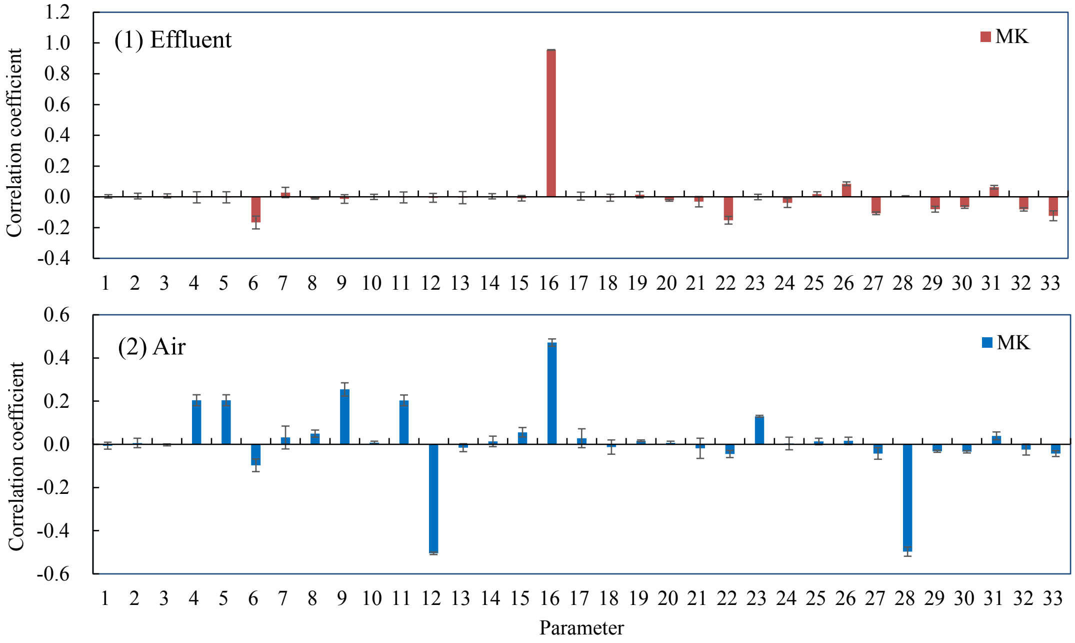

(d) MK

Figure 13 (Continue)

Figure 13 (Continue)

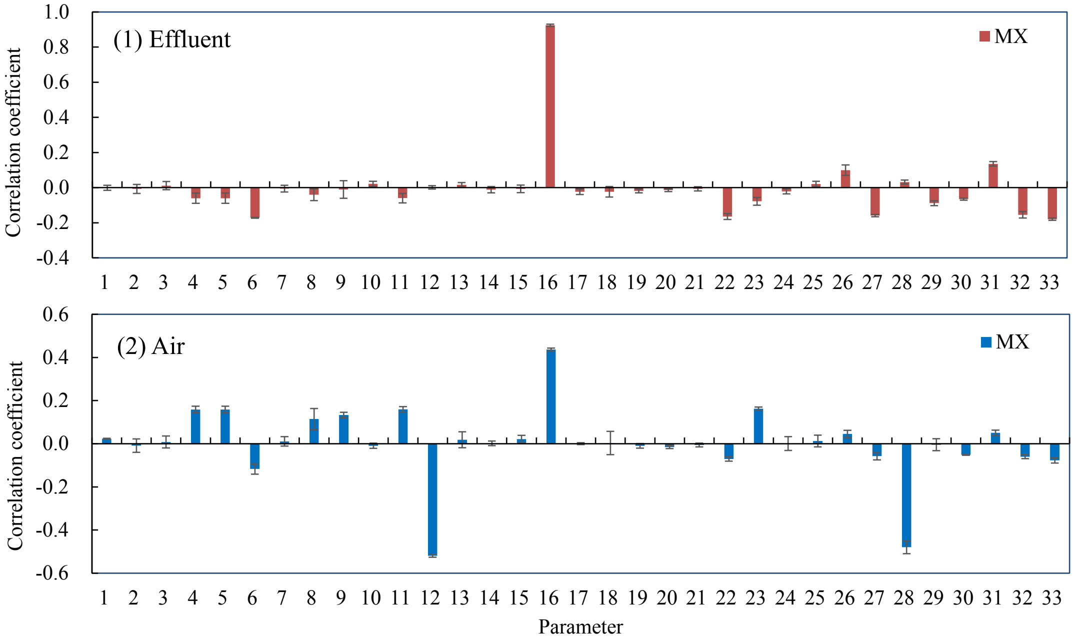

(e) MX

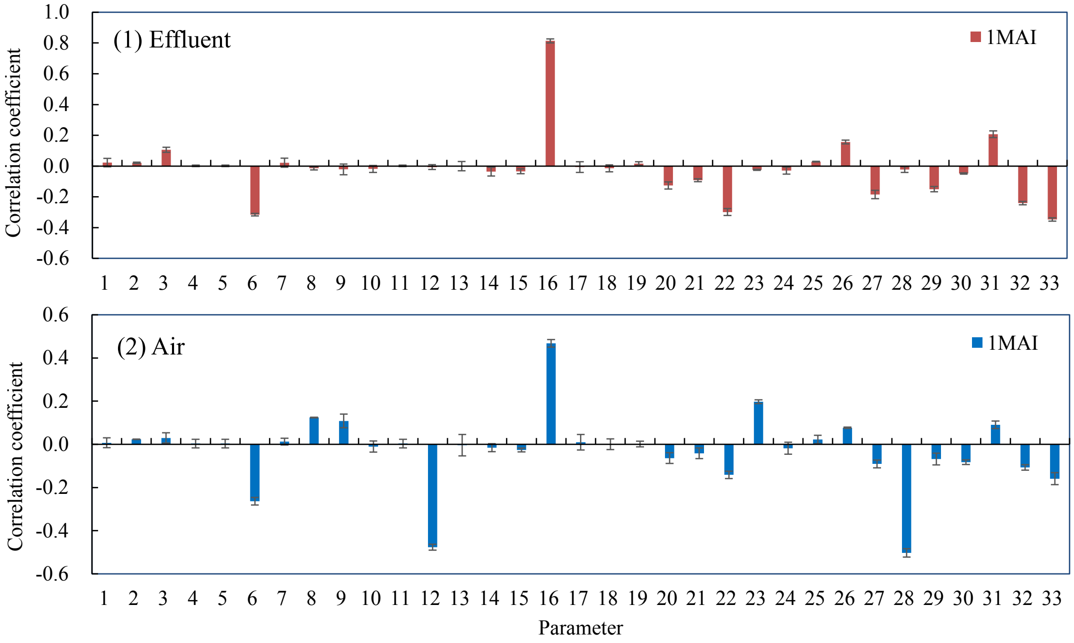

(f) 1MAI

Figure 13 (Continue)

Figure 13 (Continue)

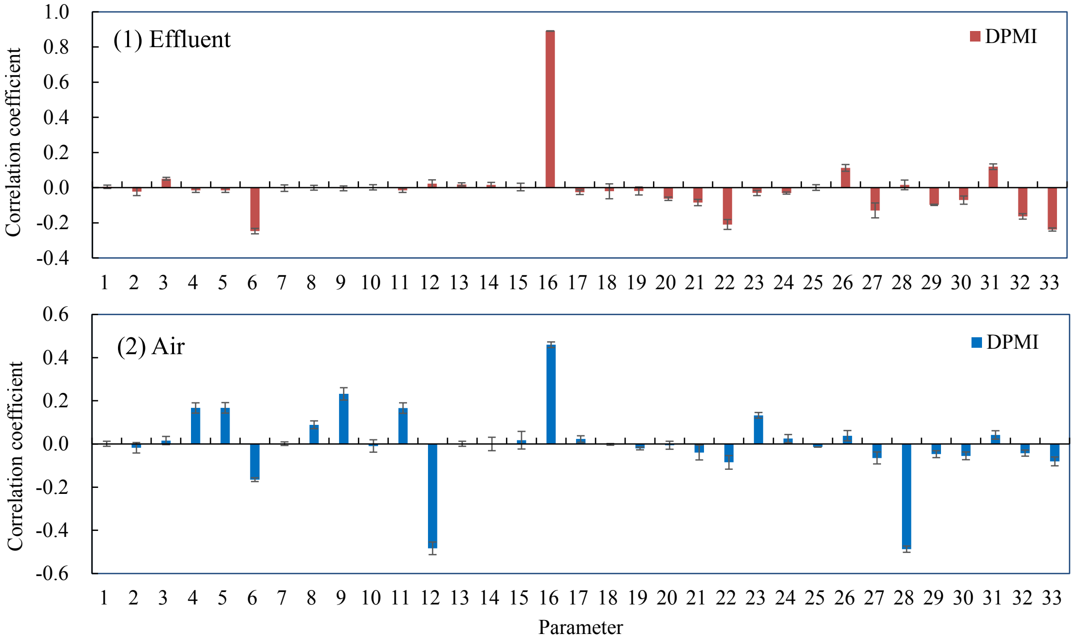

(g) DPMI

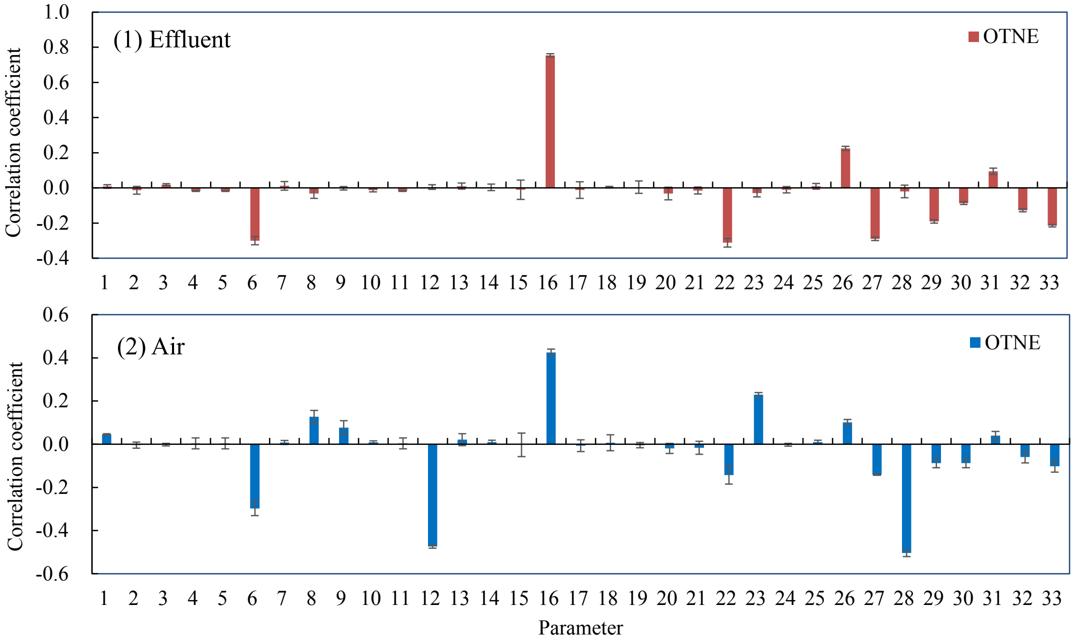

(h) OTNE

Figure 13 (Continue)

Figure 13 (Continue)

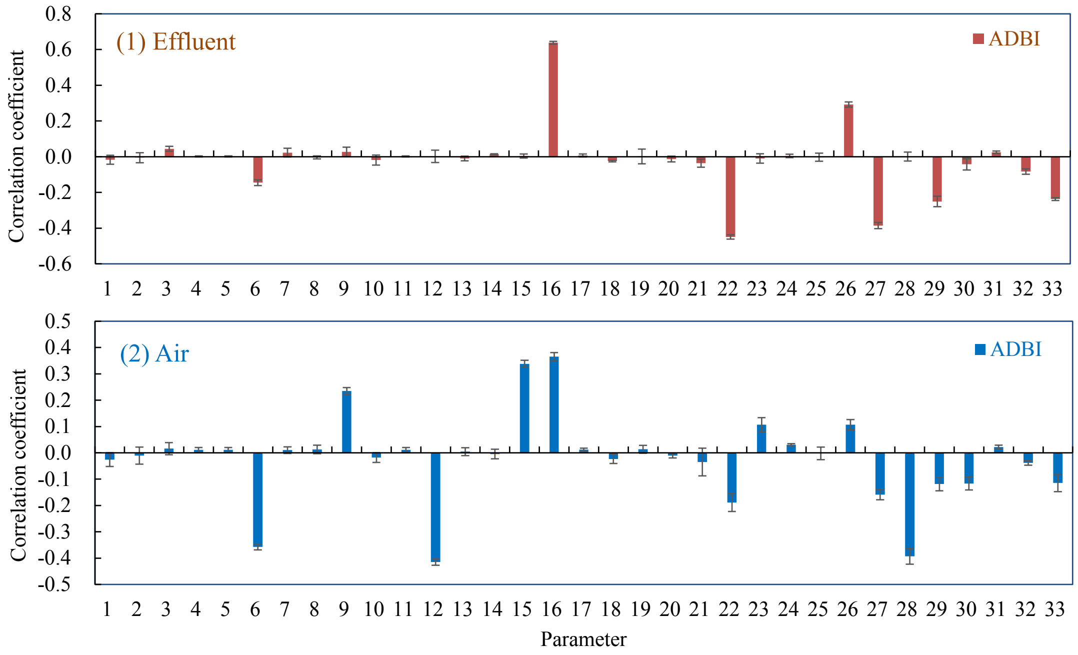

(i) ADBI

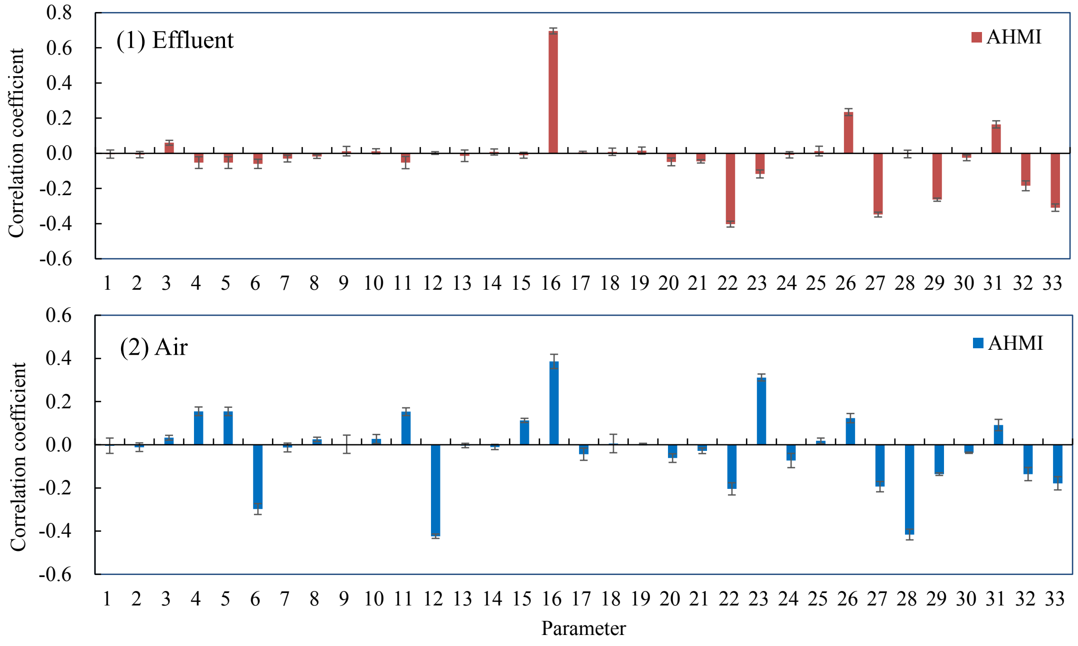

(j) AHMI

Figure 13 (Continue)

Figure 13 (Continue)

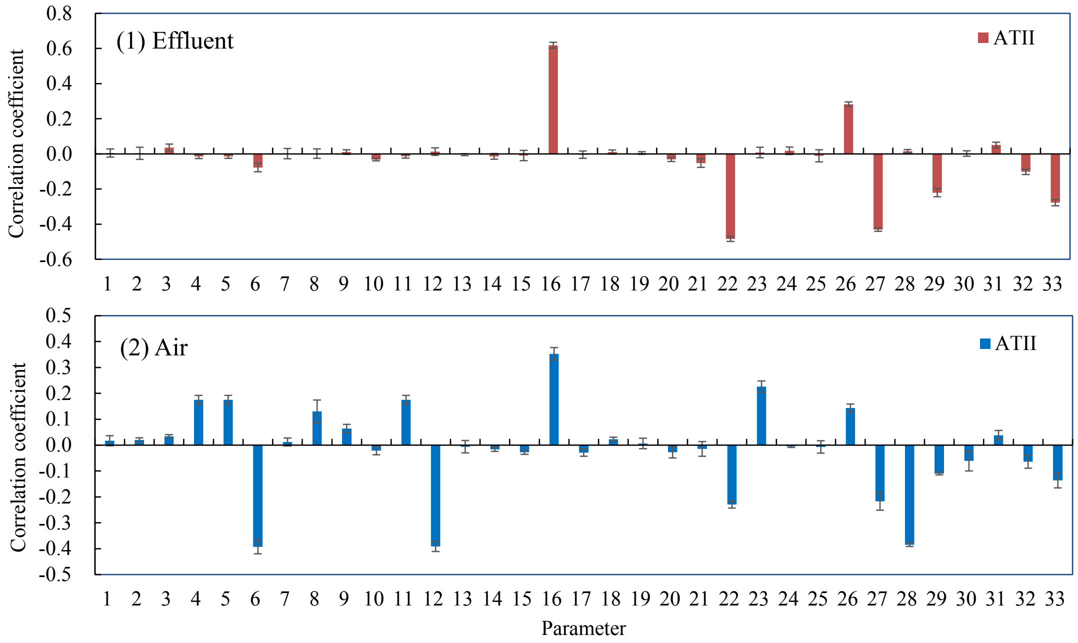

(k) ATII

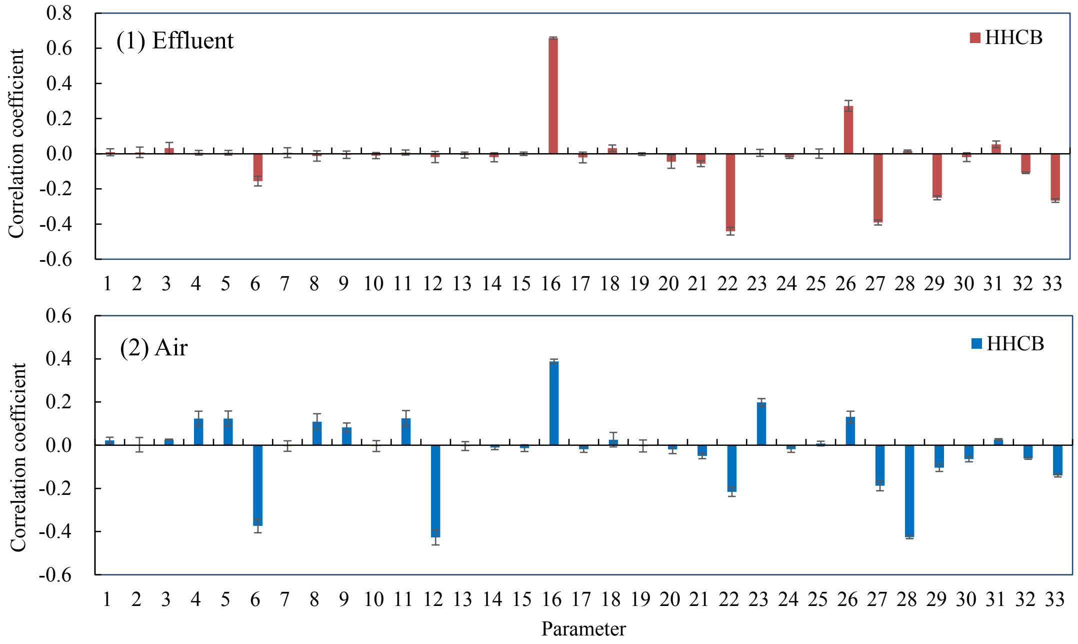

(l) HHCB

Figure 13 (Continue)

Figure 13 (Continue)

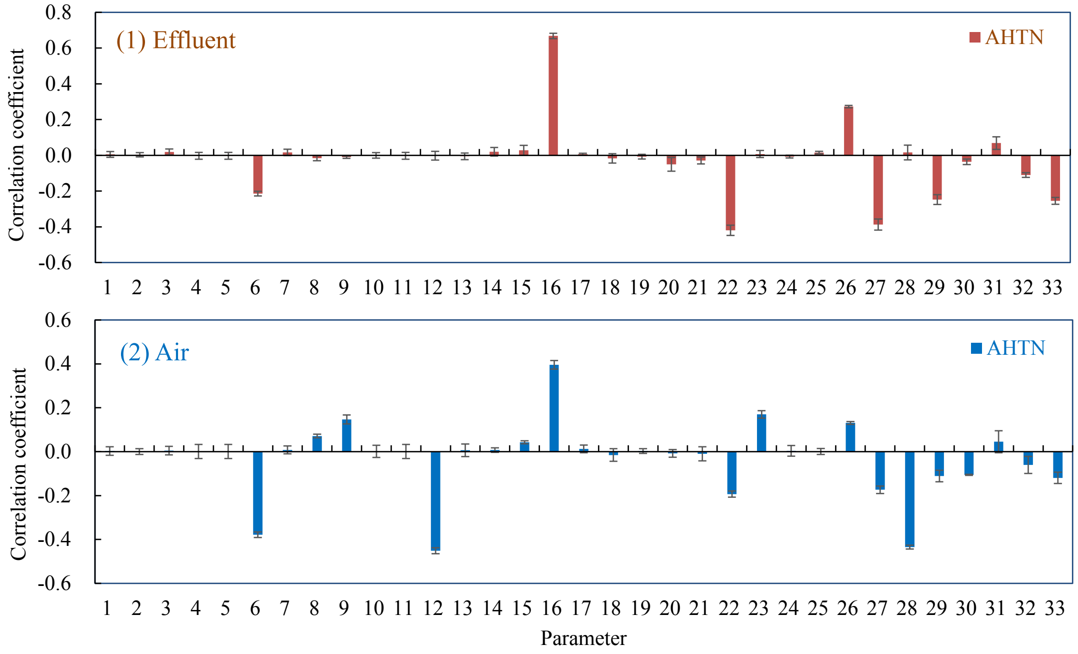

(m) AHTN

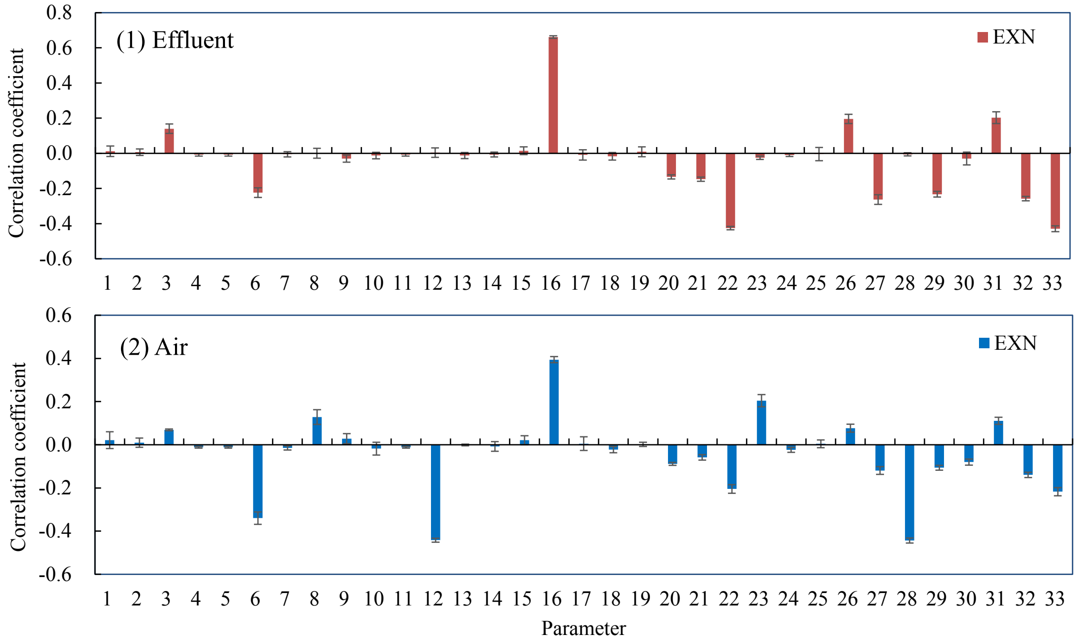

(n) EXN

Figure 13 (Continue)

Figure 13 (Continue)

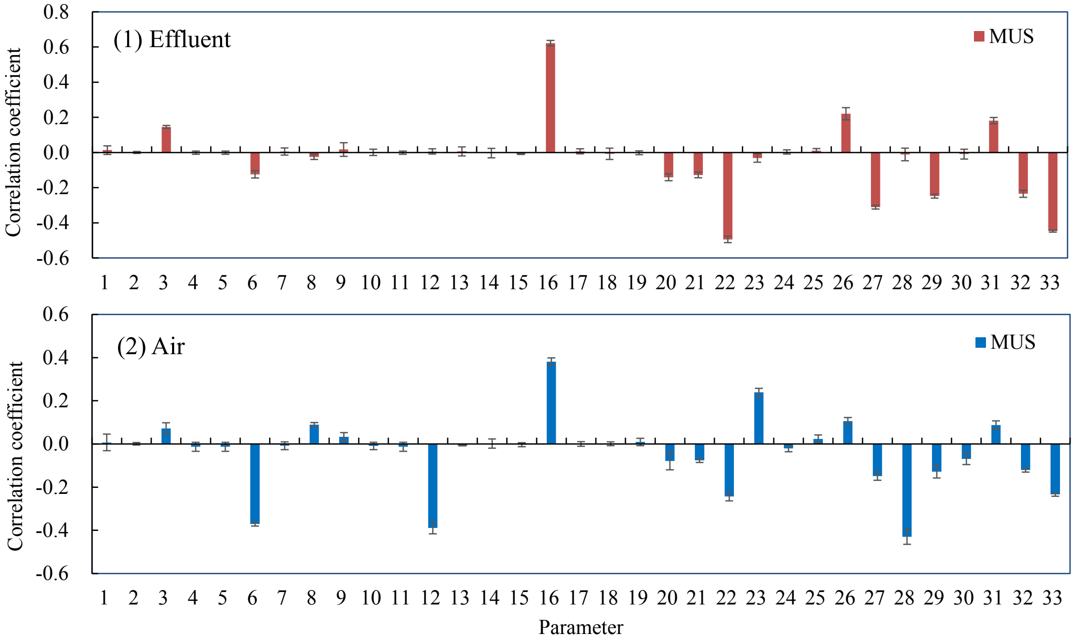

(o) MUS

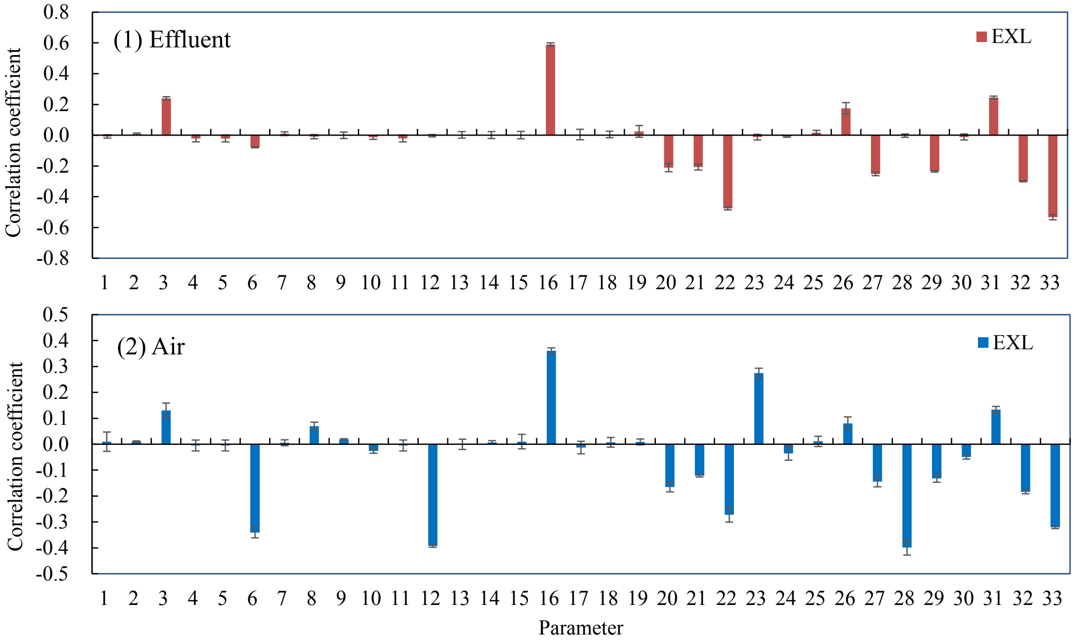

(p) EXL

Figure 13 (Continue)

Figure 13 (Continue)

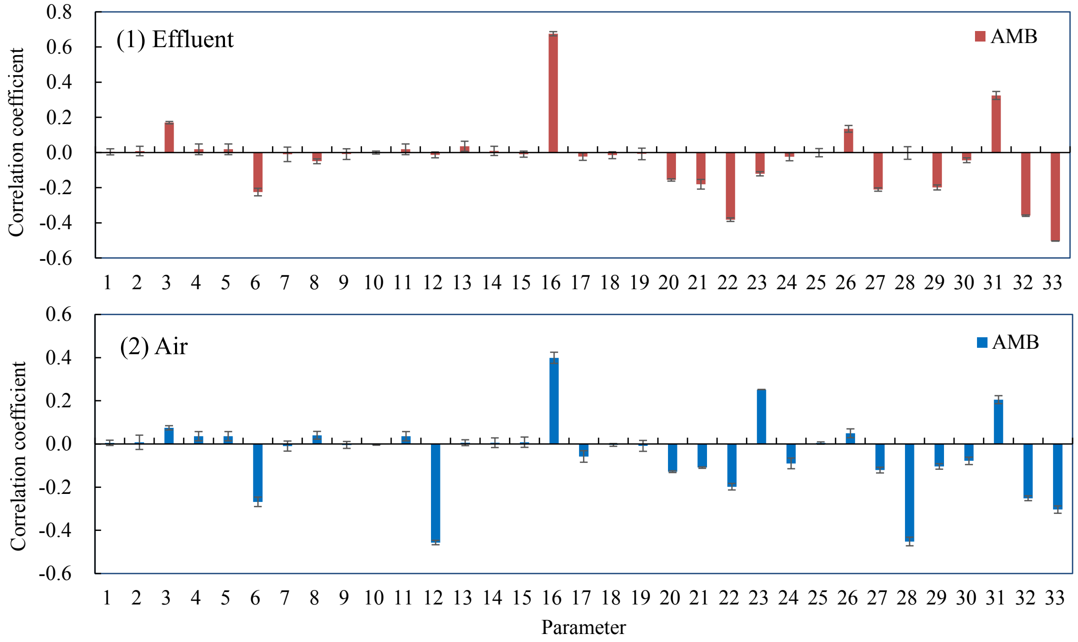

(q) AMB

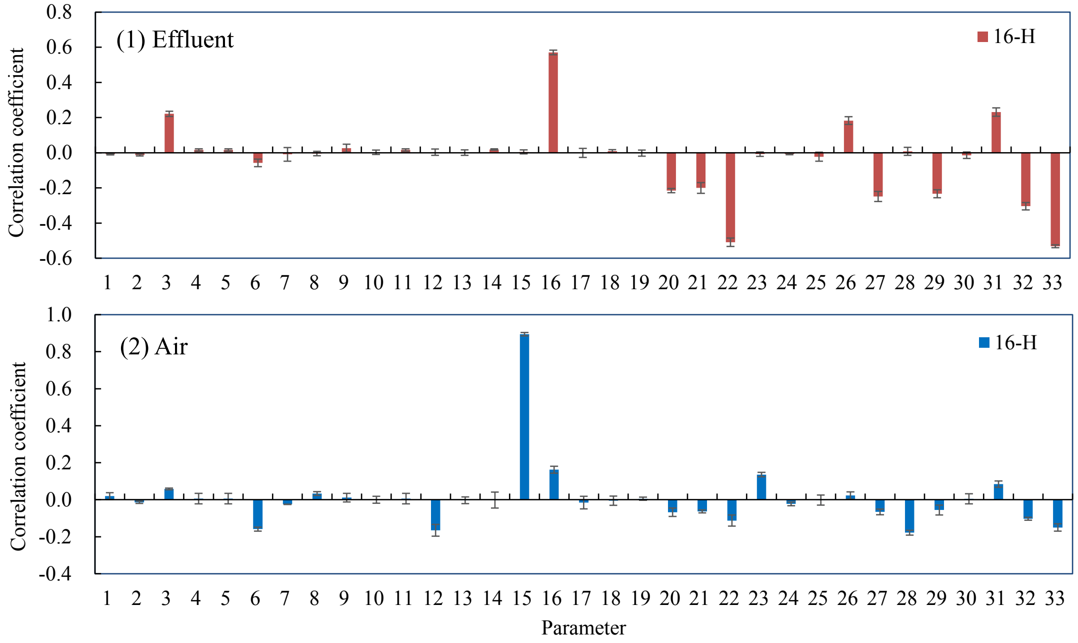

(r) 16-H

Figure 13 (Continue)

Figure 13 (Continue)

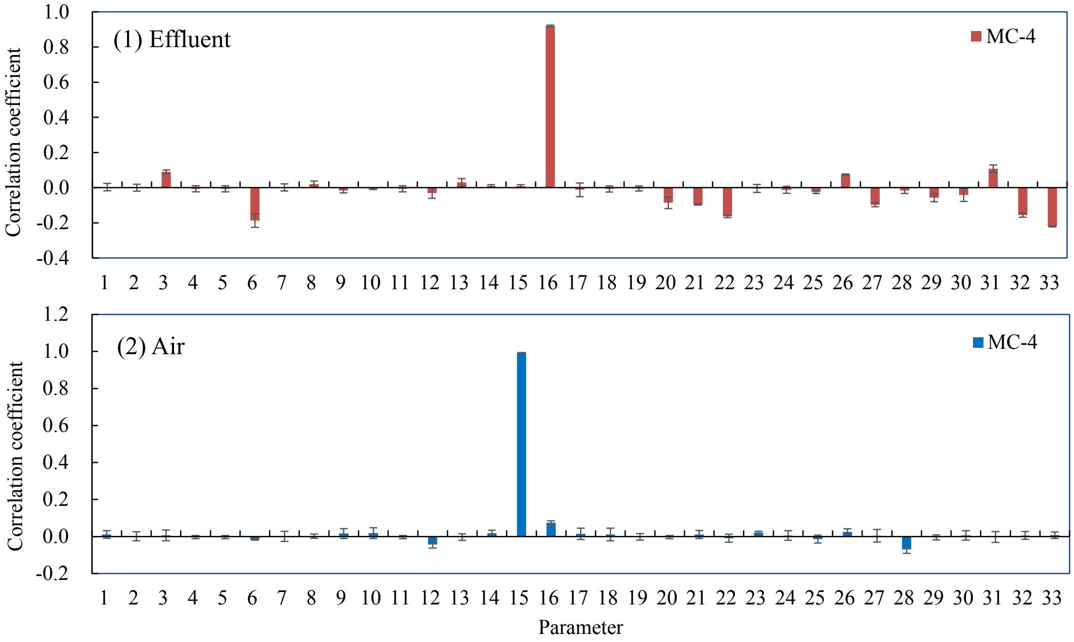

(s) MC-4

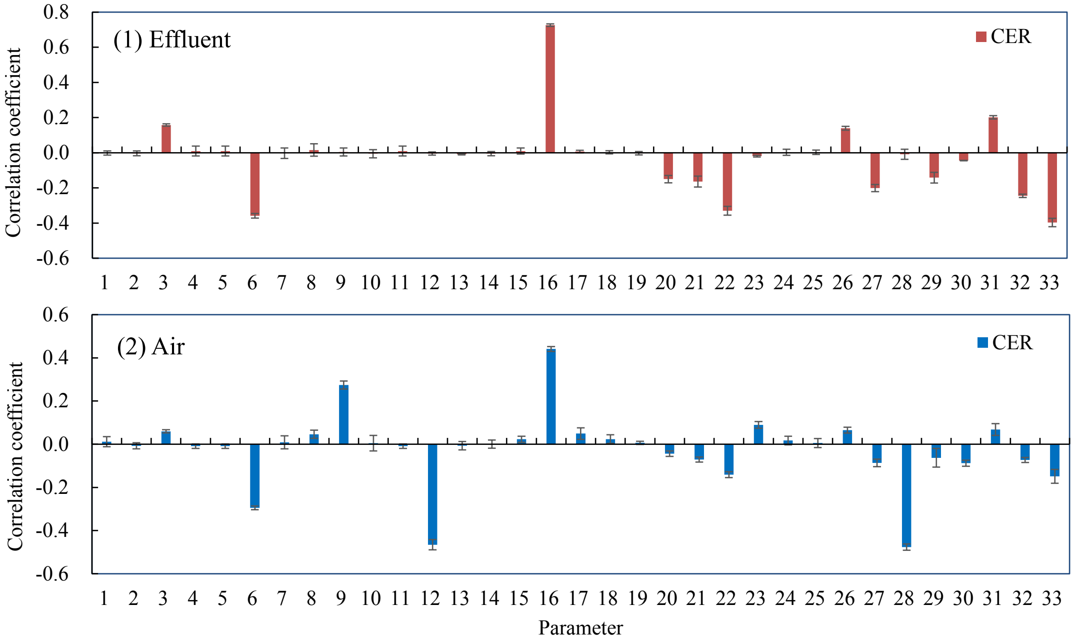

(t) CER

Figure 13 (Continue)

Figure 13 (Continue)

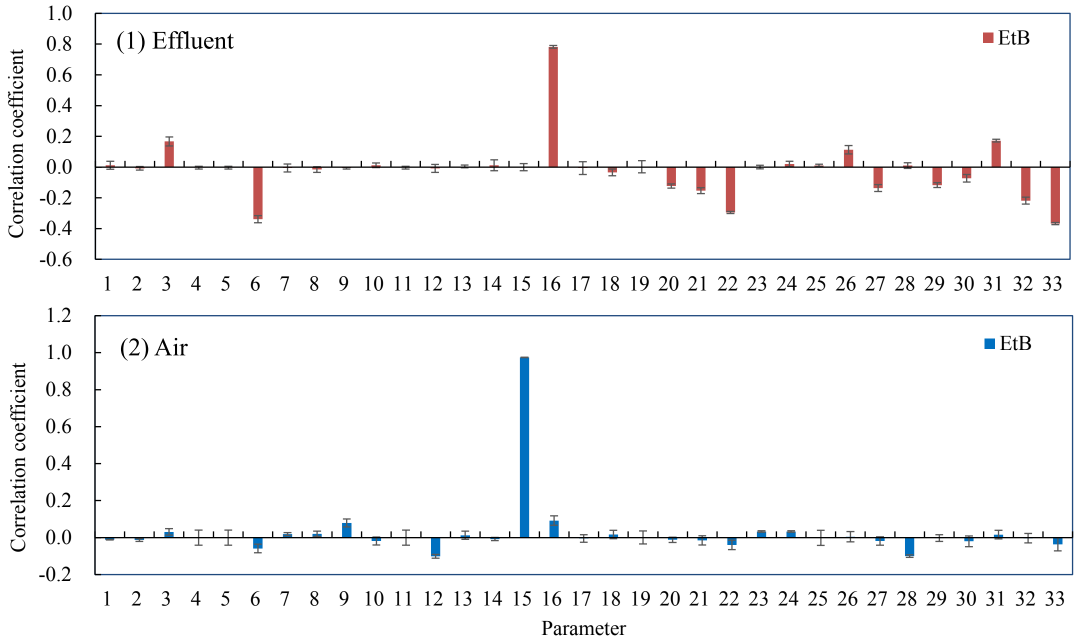

(u) EtB

Figure S13 (Continue). Sensitivity analysis of parameters using the correlation coefficient of each parameter and the output effluent and air concentrations for each SMC.

Note that the parameters considered here are 1: Half lives in air; 2: Half lives in water; 3: Half lives in biota; 4: Henry's Law constant; 5: K_{AW}; 6: K_{OW}; 7: K_{OA}; 8: Mass transfer coefficient for liquid; 9: Mass transfer coefficient for gas; 10: Aerosol deposition rate; 11: Temperature; 12: Windspeed; 13: Density of aerosol; 14: Total suspended particulate (TSP); 15: Concentration in the off-site air; 16: Concentration in the influent; 17: Area of primary tank; 18: Depth of primary tank; 19: Remove flow for primary tank; 20: Area of aeration tank; 21: Depth of aeration tank; 22: Total suspended solids (TSS) in the aeration tank; 23: Aeration rate; 24: Area for secondary tank; 25: Depth for secondary tank; 26: Recycle flow for secondary tank; 27: Remove flow for secondary tank; 28: Height of air; 29: Fraction of TSS removed in primary tank; 30: Influent TSS concentrations; 31: Influent flow rate; 32: HRT; 33: SRT.

S4. References:

1. Guerra, P.; Teslic, S.; Shah, A.; Albert, A.; Gewurtz, S. B.; Smyth, S. A. Occurrence and removal of triclosan in Canadian wastewater systems. *Environ. Sci. Pollut. R.* **2019,** *26*, (31), 31873-31886.
2. Lee, H. B.; Peart, T. E.; Sarafin, K. Occurrence of polycyclic and nitro musk compounds in Canadian sludge and wastewater samples. *Water Qual. Res. J. Can.* **2003,** *38*, (4), 683-702.
3. Guerra, P.; Teslic, S.; Shah, A.; Albert, A.; Gewurtz, S. B.; Smyth, S. A. Occurrence and removal of triclosan in Canadian wastewater systems. *Environ. Sci. Pollut. R.* **2019,** *26*, 31873-31886.
4. Paasivirta, J.; Sinkkonen, S.; Rantalainen, A. L.; Broman, D.; Zebühr, Y. Temperature dependent properties of environmentally important synthetic musks. *Environ. Sci. Pollut. R.* **2002,** *9*, (5), 345-355.
5. EPA, U. Estimation Programs Interface Suite™ for Microsoft® Windows, v 4.11 or insert version used]. United States Environmental Protection Agency, Washington, DC, USA. **2023**.
6. Campolongo, F.; Saltelli, A. Sensitivity analysis of an environmental model: an application of different analysis methods. *Reliab. Eng. Syst. Saf.* **1997,** *57*, (1), 49-69.
7. Hamby, D. A comparison of sensitivity analysis techniques. *Health Phys.* **1995,** *68*, (2), 195-204.
8. Peck, A. M.; Hornbuckle, K. C. Synthetic Musk Fragrances in Lake Michigan. *Environ. Sci. Technol.* **2004,** *38*, (2), 367-372.
9. Aronson, D.; Boethling, R.; Howard, P.; Stiteler, W. Estimating biodegradation half-lives for use in chemical screening. *Chemosphere* **2006,** *63*, (11), 1953-1960.
10. Wong, F.; Robson, M.; Melymuk, L.; Shunthirasingham, C.; Alexandrou, N.; Shoeib, M.; Luk, E.; Helm, P.; Diamond, M. L.; Hung, H. Urban sources of synthetic musk compounds to the environment. *Environ. Sci. Process. Impacts* **2019,** *21*, (1), 74-88.
11. Peck, A. M.; Hornbuckle, K. C. Synthetic musk fragrances in urban and rural air of Iowa and the Great Lakes. *Atmos. Environ.* **2006,** *40*, (32), 6101-6111.
12. Ramírez, N.; Marcé, R. M.; Borrull, F. Development of a thermal desorption-gas chromatography–mass spectrometry method for determining personal care products in air. *J. Chromatogr.* **2010,** *1217*, (26), 4430-4438.

13. Kallenborn, R.; Gatermann, R.; Planting, S.; Rimkus, G. G.; Lund, M.; Schlabach, M.; Burkow, I. C. Gas chromatographic determination of synthetic musk compounds in Norwegian air samples. *J. Chromatogr.* **1999,** *846*, (1-2), 295-306.
14. McDonough, C. A.; Helm, P. A.; Muir, D.; Puggioni, G.; Lohmann, R. Polycyclic Musks in the Air and Water of the Lower Great Lakes: Spatial Distribution and Volatilization from Surface Waters. *Environ. Sci. Technol.* **2016,** *50*, (21), 11575-11583.
15. Xie, Z.; Ebinghaus, R.; Temme, C.; Heemken, O.; Ruck, W. Air− sea exchange fluxes of synthetic polycyclic musks in the North Sea and the Arctic. *Environ. Sci. Technol.* **2007,** *41*, (16), 5654-5659.
16. Smyth, S. A.; Lishman, L. A.; McBean, E. A.; Kleywegt, S.; Yang, J. J.; Svoboda, M. L.; Lee, H. B.; Seto, P. Seasonal occurrence and removal of polycyclic and nitro musks from wastewater treatment plants in Ontario, Canada. *J. Environ. Eng. Sci.* **2008,** *7*, (4), 299-317.
